# Flexible and Accurate Substrate Processing with Distinct Presenilin/γ-Secretases in Human Cortical Neurons

**DOI:** 10.1523/ENEURO.0500-20.2021

**Published:** 2021-03-02

**Authors:** Hirotaka Watanabe, Kent Imaizumi, Tetsuo Cai, Zhi Zhou, Taisuke Tomita, Hideyuki Okano

**Affiliations:** 1 Department of Physiology, Keio University, School of Medicine, Tokyo 160-8582, Japan; 2 Laboratory of Neuropathology and Neuroscience, Graduate School of Pharmaceutical Sciences, The University of Tokyo, Tokyo 113-0033, Japan; 3 Research Fellow of Japan Society for the Promotion of Science (JSPS), Tokyo 102-0083, Japan

**Keywords:** γ-secretase, β-amyloid, iPSC, presenilin

## Abstract

Mutations in the *presenilin* genes (*PS1*, *PS2*) have been linked to the majority of familial Alzheimer’s disease (AD). Although great efforts have been made to investigate pathogenic *PS* mutations, which ultimately cause an increase in the toxic form of β-amyloid (Aβ), the intrinsic physiological functions of PS in human neurons remain to be determined. In this study, to investigate the physiological roles of PS in human neurons, we generated *PS1* conditional knock-out (KO) induced pluripotent stem cells (iPSCs), in which PS1 can be selectively abrogated under Cre transduction with or without additional *PS2* KO. We showed that iPSC-derived neural progenitor cells (NPCs) do not confer a maintenance ability in the absence of both PS1 and PS2, showing the essential role of PS in Notch signaling. We then generated *PS*-null human cortical neurons, where PS1 was intact until full neuronal differentiation occurred. Aβ40 production was reduced exclusively in human *PS1*/*PS2*-null neurons along with a concomitant accumulation of amyloid β precursor protein (APP)-C-terminal fragments CTFs, whereas Aβ42 was decreased in neurons devoid of *PS2*. Unlike previous studies in mice, in which APP cleavage is largely attributable to PS1, γ-secretase activity seemed to be comparable between PS1 and PS2. In contrast, cleavage of another substrate, N-cadherin, was impaired only in neurons devoid of *PS1*. Moreover, PS2/γ-secretase exists largely in late endosomes/lysosomes, as measured by specific antibody against the γ-secretase complex, in which Aβ42 species are supposedly produced. Using this novel stem cell-based platform, we assessed important physiological PS1/PS2 functions in mature human neurons, the dysfunction of which could underlie AD pathogenesis.

## Visual Abstract

**Figure:**
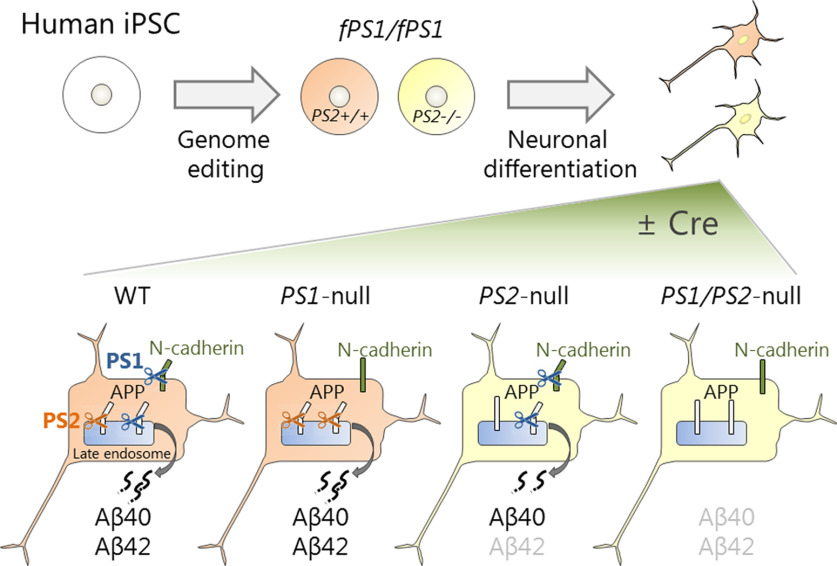

## Significance Statement

Presenilins are crucial catalytic subunits of γ-secretase, an intramembranous protease complex, whose mutations underlie Alzheimer’s disease (AD) pathogenesis via the dysregulation of β-amyloid (Aβ) generation. The γ-secretase complex exhibits heterogeneity via the assembly of PS1 or PS2, but the correlation of γ-secretase heterogeneity with substrate processing remains to be established in human neurons. Here, using a novel induced pluripotent stem cell (iPSC)-derived cellular model carrying *PS1* and/or *PS2* conditional knock-out (KO) alleles, we uncovered the unique processing of three substrates, Notch, amyloid β precursor protein (APP) and N-cadherin, by PS1 or PS2 in human neural cell contexts. Furthermore, the intrinsic subcellular localization of γ-secretase depends on PS1 or PS2, leading to putative differences in the processing of substrates. This novel platform will help ensure the correlation of γ-secretase/substrates in human neurons.

## Introduction

Alzheimer’s disease (AD) is the most common neurodegenerative dementia and is characterized by specific neuropathological lesions, including senile plaques, in the brain parenchyma of afflicted patients. The senile plaques are mainly composed of β-amyloid (Aβ) peptides and appear several decades before the onset of clinical symptoms, leading to the widely accepted amyloid hypothesis (Hardy and Selkoe, 2002). A few hundred mutations in the *amyloid β precursor protein* (*APP*) and *presenilin* (*PSEN1* and *PSEN2*, hereafter referred to as *PS1* and *PS2*) genes have been identified in familial cases of AD (FAD) to date (http://www.alzforum.org/mutations). Notably, most cases of FAD are attributed to mutations in the *PS1* and *PS2* genes, highlighting the importance of *PS* mutations in AD pathogenesis. PS1 and PS2 are expressed throughout life (Lee et al., 1996) and serve as an integral catalytic subunit of the γ-secretase complex (Kimberly et al., 2003; Takasugi et al., 2003). γ-Secretase generates Aβ by a stepwise processing of membrane-tethered APP C-terminal fragments (APP-CTFs), which are the initial ectodomain-shed products of APP by β-secretase (De Strooper et al., 1998; Vassar et al., 1999), and most FAD-linked mutations cause a relative increase in highly toxic longer Aβ species, such as Aβ42 (Borchelt et al., 1996).

Because of the presence of homologs of the *PS* and *Aph-1* genes in vertebrates, γ-secretase exhibits heterogeneity depending on which homolog is assembled into the complex. PS has two homologs, PS1 and PS2, with ∼67% sequence homology (Levy-Lahad et al., 1995; Rogaev et al., 1995; Sherrington et al., 1995) and overlapping and separate functions (De Strooper et al., 2012). In particular, PS1 is more important during development: *PS1* germline knock-out (KO) mice die perinatally, whereas *PS2* KO mice have few detectable phenotypes (Shen et al., 1997; Wong et al., 1997; Steiner et al., 1999). However, mice lacking both *PS1* and *PS2* die much earlier than *PS1* KO mice (Donoviel et al., 1999), and the neurogenesis defects in *PS1*/*PS2*-null neural progenitor cells (NPCs) are much more severe than those in *PS1*-deficient NPCs (Handler et al., 2000; Hitoshi et al., 2002; Kim and Shen, 2008). In terms of Aβ generation, PS1 and PS2 exhibit different properties in nonneuronal cells and/or nonhuman experimental systems, most of which express exogenous PS, and PS2/γ-secretase shows less total proteolytic activity than PS1/γ-secretase (Lai et al., 2003; Yonemura et al., 2011; Pintchovski et al., 2013). However, whether distinct PS/γ-secretase complexes show similar enzymatic activity in terms of substrate processing in human neurons remains unclear thus far.

Recent advancement in the induced pluripotent stem cell (iPSC) technique made it possible to access human neural cells for molecular and cellular research of neurologic disorders (Takahashi et al., 2007). Since then, substantial studies have reported many pathologic AD phenotypes using iPSC-derived neural cells from AD patients, including FAD (Penney et al., 2020). Mutations in the *PS* gene expectedly led to a significant increase in the Aβ42/40 ratio and, in some cases, tau pathology in iPSC-derived neurons (Yagi et al., 2011; Woodruff et al., 2013; Imaizumi et al., 2015; Kondo et al., 2017; Ishikawa et al., 2020; Sho et al., 2020). Despite extensive investigations, however, PS physiological functions from the aspect of PS/γ-secretase heterogeneity remain to be investigated in human neural cells.

In this study, to investigate distinct PS/γ-secretase complexes in human cortical neurons, we generated *PS1* conditional KO (cKO) iPSC lines with or without additional *PS2* KO alleles using the CRISPR/Cas9 system. We clearly demonstrated the substrate specificity between PS1/γ-secretase and PS2/γ-secretase; N-cadherin is cleaved solely by PS1, while APP/Notch is targeted by both PS1 and PS2. Moreover, using a specific antibody against the active γ-secretase complex, we showed the differences in the endogenous subcellular localization between PS1/γ-secretase and PS2/γ-secretase in human neurons for the first time. Together, these results provide direct experimental evidence showing the intrinsic heterogeneity of PS/γ-secretase in human neurons and promising insights into the molecular mechanism of PS/γ-secretase dysfunction in AD pathogenesis.

## Materials and Methods

### Culture of undifferentiated iPSCs

The healthy control human iPSC line 201B7 (female white, 36 years old; Takahashi et al., 2007) was cultured in StemFit/AK02N (Ajinomoto) as feeder-free cultures. iPSCs were passaged by 0.5 × TrypLE select (Thermo Fisher Scientific) every 7 d and seeded at 1.5 × 10^4^ cells/well in six-well plate coated with 1.5 μg/ml iMatrix-511 silk (Laminin-511 E8, Nippi) in the presence of 10 μm Y27632 (Nacalai). Culture media were changed every 2 d. For on-feeder iPSC cultures, cells were maintained on mitomycin C-treated SNL murine fibroblast feeder cells in human ESC medium: DMEM/F12 (Sigma) containing 20% KnockOut serum replacement (KSR; Life Technologies), 0.1 mm nonessential amino acids (Sigma), 0.1 mm 2-mercaptoethanol (Sigma), and 4 ng/ml fibroblast growth factor 2 (FGF-2; PeproTech) in an atmosphere containing 3% CO_2_.

### Neuronal induction

Cortical neuronal induction of iPSCs was performed as described previously with some modifications (Telezhkin et al., 2016; Sato et al., 2021), which will be published elsewhere (the patent publication number, WO/2020/045578). Briefly, semiconfluent feeder-free iPSCs were cultured for 14 d in neural medium with dual SMAD inhibitors and Wnt inhibitor. The consequent NPCs were dissociated and seeded at a density of 5 × 10^4^ cells/cm^2^ on multiwell plate coated with poly-ornithine and Matrigel (Corning). Terminal differentiation was induced in neural medium supplemented with B27 (Invitrogen) and 10 μm DAPT (Sigma) for 6 d. After day 6, the culture was maintained in BrainPhys Neuronal Medium (Stemcell Technologies) supplemented with 10 ng/ml BDNF, 10 ng/ml GDNF, 200 μm ascorbic acid, 0.5 mm dbcAMP, and changed medium every 3–4 d with a half volume until day 45.

### Generation of conditional PS1 KO and PS2 KO iPSCs

To construct a targeting vector for the *PS1* allele, a 4.41-kb genomic DNA, which contains the exons 2–3, was first amplified by PCR using genomic DNA of 201B7 iPSC as a template, with a primer pair 5′-CCTGGCCTCAAGTAGTAACACCCAT-3′ and 5′-CACAGCAGCCCACAAAAGGAAAACT-3′, and subcloned into pCR-BluntII TOPO (Invitrogen). After sequence confirmation, a 1.18-kb NdeI-NotI DNA fragment (L1), a 1.12-kb NotI-*Sbf*I fragment (L2) encompassing the second and third exon of the *PS1* gene, and a 1.08-kb *BamH*I/SacII-*BamH*I fragment (R1) were amplified by PCR using the above genomic fragment as a template. The PCR primers are 5′-TACCATATGAGTCTCACTCTGTTGCCCAGG-3′ and 5′-ATTGCGGCCGCAGGACGGACAGATAACATG-3′ for the left arm L1 (the underlined sequences are for NdeI and NotI), 5′-TAAGCGGCCGCGGGCATTGTGATAAGG-3′ and 5′-ATGCCTGCAGGAACCCTTAGAACTTCTACAC-3′ for the left arm L2 (the underlined sequences are for NotI/SacII and *Sbf*I), and 5′-ATTGGATCCGCGGAGGGATTCAGGAAAAGAAC-3′ and 5′-ATTGGATCCTGGGCACATCAAAACTTCC-3′ for the right arm R1 (the underlined sequences are for *BamH*I/SacII and *BamH*I). These genomic fragments were used as 5′ (L1 and L2) and 3′ (R1) homologous regions in the *PS1* targeting vector. Both L1 and L2 fragments were subcloned into NdeI-*Sbf*I sites in the pUC-FRT-PGK-neo-FRT plasmid (#519), in which PGK-neoΔtk (a fusion protein of a neomycin-phosphotransferase and a truncated version of herpes simplex virus type 1 thymidine kinase) cassette was flanked by two FLP recognition target (*FRT*) sites to allow removal of the neoΔtk gene by FLP, as its presence can suppress transcription of the target gene. After sequence confirmation of homologous arm region, a *loxP* fragment was inserted into a SacII site in the left arm, which was a vicinity of the junction between L1 and L2 fragments. A *loxP* fragment was generated by annealing the following oligonucleotides: 5′-ATCGATATAACTTCGTATAGCATACATTATACGAAGTTATTTGC-3′ and 5′-AAATAACTTCGTATAATGTATGCTATACGAAGTTATATCGATGC-3′ (a 34-nt *loxP* sequence is underlined). Next, a right arm fragment (R1) was subcloned into *BamH*I site of the left-arm plasmid and confirmed by sequencing. A *loxP* fragment was also inserted into a SacII site in the right arm to generate the *PS1* targeting vector (neo). PGK-neoΔtk cassette was replaced with PGK-puΔtk (a fusion protein of a puromycin N-acetyltransferase and a truncated version of herpes simplex virus type 1 thymidine kinase) by Gateway BP reaction to generate the *PS1* targeting vector (puro). To generate a plasmid expressing single guide RNA (sgRNA) for *PS1* genome editing, the following oligonucleotides were annealed and inserted into a *Bbs*I site of pSpCas9(BB)-2A-GFP (PX458), which can simultaneously express *Streptococcus pyogenes* Cas9 (SpCas9) and GFP: 5′-CACCGTTATCTGTCCGTCCTGCCTT-3′ and 5′-AAACAAGGCAGGACGGACAGATAAC-3′ for *PS1* sgRNA1, and 5′-CACCGTAGAAGTTCTAAGGGTTCAA-3′ and 5′-AAACTTGAACCCTTAGAACTTCTAC-3′ for *PS1* sgRNA2 (the targeted *PS1* sequences are underlined).

To generate iPSCs carrying the targeted/floxed *PS1* alleles, 201B7 iPSCs were electroporated with a targeting vector (puro) along with two sgRNA-expressing plasmids. Puromycin was applied to the culture at 0.5 μg/ml 24 h later and the surviving iPSC clones were picked following 7 d of puromycin selection. Genomic DNAs around the targeting region of total 12 iPSC clones were first amplified by PCR using the primer pair for *PS1* genomic cloning as shown above, which encompasses both homologous recombination arms. Next, nested PCR was performed using the primer pair 5′-CTCCTGGCTGAGTCTGCGAT-3′ and 5′-AGAACCGCCTGAGACACCAA-3′, which encompasses the upstream *loxP* sequence. Two iPSC clones were positive for proper homologous recombination in one allele, giving rise to 345- and 392-bp bands, which represent the wild type (WT) and the targeted allele, respectively. This heterozygous clone was further electroporated with a targeting vector (neo) along with two sgRNA-expressing plasmids. G418 was applied to the culture at 100 μg/ml 24 h later and the surviving iPSC clones were picked following 10 d of G418 selection and screened as previously. Two out of 30 iPSC clones were confirmed to carry the correct homologous recombination at both alleles. We verified proper recombination by sequencing to confirm the presence of the single *loxP* sites in targeted allele. To generate iPSCs bearing *floxed* and *deleted PS1* alleles, these homozygous targeted *PS1* clones (#245 and #249) were transfected with pCAGGS-FLP and pCAGGS-Cre, respectively, and selected under 10 μm ganciclovir treatment for 10 d. In this study, most experiments were conducted using one targeted *PS1* clone (#245)-derived cells.

To create *fPS1*/*fPS1*;*PS2*^−/−^ iPSC, *PS1* floxed iPSC clone was transfected with two crRNAs, tracrRNA and SpCas9 proteins to delete the exon 5 in the *PS2* gene. Alt-R CRISPR-Cas9 crRNA (IDT) sequences are as follows: 5′-CCGGCCCUGACUGCUCCUCGGUUUUAGAGCUAUGCU-3′ for *PS2* crRNA1, and 5′-CUACAAGUACCGCUGCUACAGUUUUAGAGCUAUGCU-3′ for *PS2* crRNA2 (the targeted *PS2* sequences are underlined). Twelve iPSC clones were picked and expression of PS2 was screened by Western blot analysis, then PS2 signals were negative in two clones (clones #10 and #12). We verified proper disruption of the *PS2* gene by sequencing to confirm deletion and inversion around the exon5 in the *PS2* alleles of clone #12, respectively.

### Colony-forming assay

Colony-forming assay using iPSCs was performed as described previously (Imaizumi et al., 2015; Fujimori et al., 2017). Briefly, iPSCs were pretreated for 6 d with 3 μm SB431542 (Tocris) and 150 nm LDN193189 (StemRD). To make clonal neurospheres, the cells were then dissociated and seeded at a density of 10 cell/μl in media hormone mix (MHM) with selected growth factors and inhibitors under hypoxic conditions. The growth factors and inhibitors included 20 ng/ml FGF-2, B27 supplement (Invitrogen), 2 μm SB431542 and 10 μm Y-27632 (Calbiochem). We infected lentiviruses expressing either mCherry-nls-Cre or mCherry-nls-ΔCre at multiplicity of infection (MOI) ≈1 just at the beginning of the culture. Here, we defined the day on which neurosphere culture was started as day 0. On day 12, primary neurospheres were then dissociated and seeded at a density of 100 cell/μl to make secondary neurospheres. For flow cytometry analysis, dissociated neurospheres were suspended in PBS supplemented with 4% fetal bovine serum (FBS) and analyzed mCherry fluorescence with a FACSAria (BD Biosciences) using an 85-μm nozzle, to calculate the lentivirus infected population. On day 18, secondary neurospheres were then dissociated and the fluorescence of the cells was analyzed by flow cytometry.

### Quantitative reverse transcription-PCR (qRT-PCR)

RNA extraction and qRT-PCR were performed as described previously (Imaizumi et al., 2015; Fujimori et al., 2017). Briefly, total RNA was purified with RNeasy Micro kit or RNeasy Mini kit (QIAGEN) and reverse-transcribed in the presence of random hexamers. qPCR reactions were performed using TB Green Premix Ex Taq II (TAKARA BIO Inc.) in ViiA 7 System (Applied Biosystems) with cDNA and gene-specific primers. Analysis was performed with at least three independent cultures and threshold cycle (Ct) values of interest were normalized to *GAPDH*. The primer pairs used in this study are as follows: 5′-ATCTGGGAGCCTGCAAGTGAC-3′ and 5′-ACAGAAAACAAAGCCTCTTGAGGT-3′ for *PS1*, 5′-TGCATGATCGTGGTGGTAGC-3′ and 5′-GTCCTCAGTGAATGGCGTGT-3′ for *PS2*, 5′-ACAGGTGGCCTTAAGAACTTCAT-3′ and 5′-CCACCTGGTTCCGTACAGAC-3′ for *Nicastrin*, 5′-CAGGTGTGGTTGGGATCCA-3′ and 5′-GGAGCAGGATAATGGCTGCT-3′ for *Aph-1A*, 5′-GCTGATCTTTGGAGCGTTTGTC-3′ and 5′-ACTCTTCAAACCTTCACTGGCT-3′ for *Aph-1B*, 5′-TCCTTGTCCCAGCCTACACA-3′ and 5′-AGCACTATCACCCAGAAGAGGA-3′ for *PEN-2*, 5′-ATCACCATCCTTCCGCAGCA-3′ and 5′-AACAGTGCCCGTGGATGACT-3′ for *BACE1*, 5′-TGCAGATGGGAGTGAAGACAA-3′ and 5′-TCCTCGTCATCATCGGCTTC-3′ for *APP*, 5′-CCTGTGTTAAGCGGAAAACC-3′ and 5′-AGAGACTTTGTCCTTTGCCTGT-3′ for *MAP2*, 5′-AGCAATGCCTACCTGAGTGA-3′ and 5′-AGCTGCTGTGACTGATCTCA-3′ for *PSD95*, 5′-TTTGTCACCGTGGCCGTGTTT-3′ and 5′-CGTGGCCAGAAAGTCCAGCAT-3′ for *Synaptophysin*, 5′-GAACGGGGCTAACAAAGATATGCA-3′ and 5′-GATGTCCCGGTTGGCAAAGTG-3′ for *NOTCH1*, 5′-TTGTGTCTCGACCCTGCCTGAA-3′ and 5′-ACAGGCAGGCATCCGTCCATT-3′ for *NOTCH2*, 5′-AAAAGACGAAGAGCAAGAATA-3′ and 5′-GCTTCACTGTCATTTCCAGAATGT-3′ for *HES1*, 5′-CGCATCAACAGCAGCATCGAG-3′ and 5′-CGACGAAGGCTTTGCTGTGC-3′ for *HES5*, 5′-AGTTTGTGCCAGGGTTTTTG-3′ and 5′-ACTTCACCTTCCCTCCAACC-3′ for *OCT4*, 5′-ACCACACCGGTTTCCTCCTTCACA-3′ and 5′-TTGCCATGGTGAAGCTGGGCAT-3′ for *PAX6*, 5′-CACCATTGGCAATGAGCGGTTC-3′ and 5′-AGGTCTTTGCGGATGTCCACGT-3′ for *ACTB*, 5′-AGGCTGAGAACGGGAAGCTT-3′ and 5′-ACTCCACGACGTACTCAGCG-3′ for *GAPDH*.

### Lentivirus production and infection

pCAG-HIVgp48 and pCMV-VSVG-RSV-Rev are plasmids encoding the gag/pol/tat proteins and the pseudotyped envelope of lentivirus, respectively. pFUGW-EGFP-nls-Cre and pFUGW-EGFP-nls were previously described (Watanabe et al., 2009). pFUGW-mCherry-nls-Cre and pFUGW-mCherry-nls were generated by replacing the *EGFP* gene in pFUGW-EGFP-nls-Cre and pFUGW-EGFP-nls with the *mCherry* gene. For self-inactivating lentivirus vectors expressing human *PS1*, human *PS2* and mouse *PS2* under the *EF-1α* promoter, each cds was first subcloned into pENTR/D-TOPO vector (Invitrogen) and then verified by sequencing. The respective *PS* cds on the pENTR vector was subcloned into pCSII-EF-Rfa-IRES2-Venus plasmid (RIKEN BRC #RDB04389) by GATEWAY technology (Invitrogen). *PS* cds fragments were amplified from brain cDNAs using specific primers as follows (the start codon sequences are underlined): 5′-CACCATGACAGAGTTACCTGCACC-3′ and 5′-AGTATTTCTATACAGTTGCTCC-3′ for human *PS1* (1433 bp), 5′-CACCATGCTCACATTCATGGCCTC-3′ and 5′-ACACCATGTCCCTCAGATGTAG-3′ for human *PS2* (1359 bp), 5′-CACCATGCTCGCATTCATGGCCTC-3′ and 5′-TCCGCCTGGCTCCTGTCAGATG-3′ for mouse *PS2* (1362 bp).

Production of recombinant lentiviruses is achieved by transfecting HEK293T cells with three plasmids. Lentiviruses were harvested 48 h after transfection by collecting the medium from transfected cells, and filtrated with a 0.45-μm filter. Titer of the lentivirus was estimated by measuring the EGFP-positive or mCherry-positive cells with fluorescent microscopy, following the infection of diluted lentivirus to HEK293T cells. Neurons were infected with each lentivirus at ∼3 of MOI.

### PS DKO MEF cultures and Aβ production assay

Stable mouse embryonic fibroblasts that lack both *PS1* and *PS2* and express human *APP* gene carrying Swedish mutation heterologously (DKONL; Herreman et al., 2000; Watanabe et al., 2005) were maintained in high glucose DMEM (Invitrogen) supplemented with 10% FBS and 0.1 μg/ml puromycin. DKONL cells were infected with *PS*-expressing lentivirus and the culture medium was changed 1 d after lentivirus infection. The culture medium was collected following 48 h incubation and spun down for removing cell debris and stored at −80°C until use for Aβ ELISA.

### Western blot analysis

Cultured neuronal cells at six to eight weeks were homogenized in RIPA buffer [50 mm Tris-Cl (pH 7.6), 150 mm NaCl, 0.5 mm EDTA, 1% NP40, 0.5% sodium deoxycholate, 0.1% SDS, Complete protease inhibitor cocktail (Roche), 1 mm PMSF]. Equal amount (10–20 μg per lane) of proteins were separated in Extra PAGE One Precast Gel (Nacalai tesque Inc.) and transferred to PVDF membranes. The membranes were blocked in 5% nonfat milk/TBS for 1 h, and incubated with specific primary antibodies shown as below: rabbit anti-PS1 NTF (1:1000, G1Nr5; Sato et al., 2008), rabbit anti-PS1 CTF (1:1000, G1L3; Tomita et al., 1999), rabbit anti-PS2 CTF (1:5000, #ab51249, abcam), rabbit anti-APP (1:200, #18 961, IBL), mouse anti-N-cadherin (1:1000, #610920, BD Transduction), mouse anti-phospho tau (1:500, #MN1020, Millipore), rabbit anti-tau (1:1000, #A0024, DAKO), mouse anti-actin (1:10 000, #A1978, Sigma). The membrane was then incubated with IRDye 800CW or IRDye 680-labeled secondary antibodies (LI-COR Bioscience). Signals were developed and quantified with an Odyssey Infrared Imaging System (LI-COR Bioscience).

### Immunocytochemistry

iPSC-derived neuronal cultures in multiwell plate from around day 45 were fixed with methanol or 4% paraformaldehyde, blocked with a solution containing 3% nonfat dry milk and 0.1% saponin for 1 h at room temperature, and incubated with the indicated primary antibodies overnight at 4°C. The following primary antibodies were used: mouse anti-NeuN (1:200, #MAB377, Millipore), rabbit anti-TBR1 (1:200, #ab31940, abcam), mouse anti-glycosylated nicastrin (1:200, A5226A; Hayashi et al., 2012), rabbit anti-APP (1:500, #ab32136, abcam), rabbit anti-MAP2 (1:200, #AB5622, Millipore), mouse anti-βIII-Tubulin (1:500, #T8660, Sigma), mouse anti-LAMP1 (1:100, #328602, BioLegend), and mouse anti-EEA1 (1:100, #610456, BD Transduction). Cultures were then washed in phosphate-buffered saline and incubated with appropriate Alexa Fluor (405, 488, 555, or 647)-conjugated secondary antibodies (Invitrogen) for 1 h at room temperature. Signals were detected by LSM 700 confocal microscope (Carl Zeiss).

For immunofluorescent analysis, 96-well plates were imaged with an IN Cell Analyzer 6000 high-content cellular analysis system (GE Healthcare). A set of 3 × 3 fields was collected from each well using the 20× objective lens. Comprehensive analysis was performed with IN Cell Developer Toolbox version 1.9 (GE Healthcare). First, the lentivirus (ΔCre or Cre)-infected neuronal population was identified by signals for mCherry and MAP2 fluorescence, which were defined as mCherry^+^ nuclei that were >50 μm^2^ and with intensity levels that were appropriate brightness of intact cells. In each traced MAP2+ region, puncta positive for glycosylated nicastrin and LAMP1 (late endosomes/lysosomes) or EEA1 (early endosomes) were detected; from these images, the fluorescence area of each puncta and their colocalization was measured and analyzed.

### ELISAs for Aβs

iPSC-derived neurons were differentiated by plating almost the same number of NPCs and cultures were maintained in 48-well plate until analysis (day 45). Medium was fully changed with 500 μl/well of fresh medium 48 h before the harvest. The collected medium was centrifuged to remove insoluble material and stored at −80°C until analysis. The remaining neuronal cells were lysed in RIPA buffer and protein concentration was measured by BCA Protein assay (Pierce). Aβ40 and Aβ42 levels in the conditioned medium were measured using commercial kits, Human βAmyloid (1–40) ELISA kit II (catalog #298-64601) and Human βAmyloid (1–42) ELISA Kit High Sensitive (catalog #296-64401) from Wako, respectively, according to the manufacturer’s protocol. Each Aβ concentration was normalized by protein levels of the culture.

### Statistical analysis

**Figure 1.**
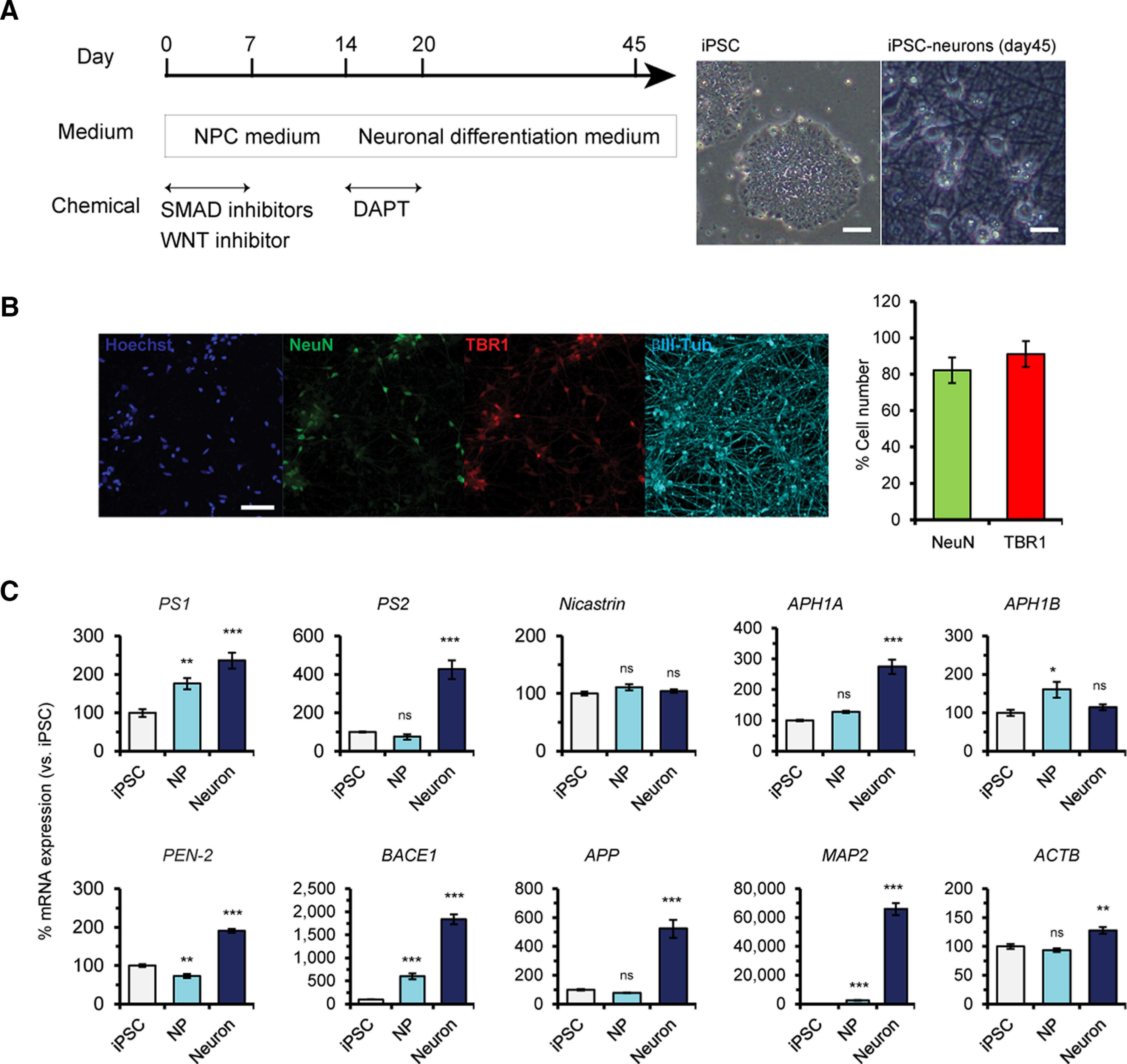
Expression of genes encoding integral γ-secretase subunits during cortical neuronal differentiation of a healthy individual-derived iPSC line, 201B7. ***A***, Strategy of cortical neuron differentiation from iPSCs. Representative images of iPSC colonies (left) and iPSC-derived cortical neurons (right) are also shown. Scale bars: 100 μm (iPSC) and 20 μm (iPSC-neurons). ***B***, Representative images of iPSC neurons stained with antibodies specific for NeuN, TBR1, and βIII-tubulin are shown. Scale bar: 50 μm. Numbers of NeuN+ or TBR1+ cells among Hoechst 33342-stained cells were calculated. Data represent the mean ± SEM (*n* = 3 of independent culture batches). ***C***, qRT-PCR analysis of iPSCs (day 0), NPCs (NP; day 14), and iPSC-derived cortical neurons (neuron; day 45). Several genes encoding γ-secretase subunits, such as *PS1*, *PS2*, *APH-1A*, and *PEN-2*, were upregulated in iPSC neurons compared with iPSCs and NPCs. *BACE1* and *APP* were also upregulated in iPSC neurons. Data represent the mean ± SEM (*n* = 3–4 of independent culture batches). ns, not significant; *p <* *0.05, **p <* *0.01, ***p <* *0.001 by Dunnett’s test versus iPSCs.

**Figure 2.**
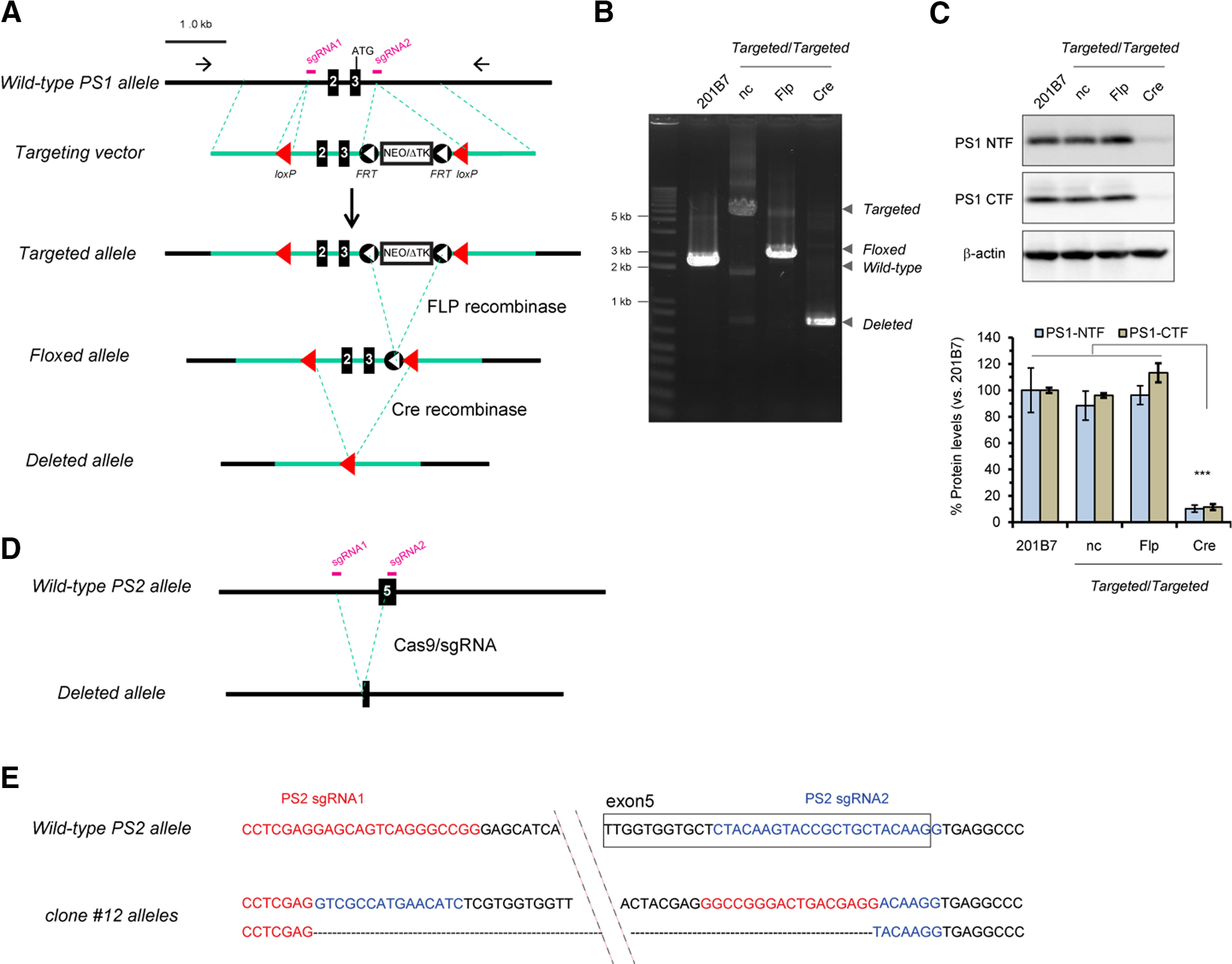
Generation of *PS* cKO iPSCs. ***A***, Targeting strategy for *PS1* cKO. In the targeting vector, exons 2/3 are flanked by a *loxP* site (red arrowhead) and a *PGK-neo* (or *PGK-puro*) selection cassette followed by a *loxP*. The *neo* selection cassette is flanked by two *FRT* (FLP recognition target) sites (black circles), so that the cassette can be removed by FLP recombinase. Green lines were the genomic sequences of homologous regions. iPSC clones were screened by genomic PCR using the primer pairs (arrows) to discriminate iPSC cells carrying the proper recombination from untargeted ones. The homozygous targeted clones were transfected with a plasmid expressing *Flp* gene to generate homozygous floxed *PS1* iPSC. In the presence of Cre, the floxed allele will be recombined to produce the deleted *PS1* allele. ***B***, Genomic PCR for respective *PS1* alleles. Genomic PCR was performed using primer pair encompassing two *loxP* sites, following transfection with plasmid expressing *Flp* or *Cre* recombinase. Parental iPSC line (201B7) and transfectant with empty vector (nc) were also shown. ***C***, PS1 protein expression in iPSC clone carrying respective *PS1* allele. Quantitative analysis shows nearly eliminated levels of PS1 proteins in total lysates isolated from the iPSC clone carrying deleted allele after *Cre* transduction, compared with other iPSC clones, using rabbit polyclonal antibodies specific for PS1-NTF and PS1-CTF. Data represent the mean ± SEM (*n* = 3 of independent culture batches). ns, not significant; ***p <* *0.005 by Tukey’s test among the genotypes. ***D***, KO strategy for *PS2* gene. sgRNAs were transfected along with Cas9 proteins, to delete the genomic region around the exon 5. ***E***, Sequence of both alleles around the *PS2* targeted region from clone #12 (nearly negative for PS2 protein was confirmed by PS2 Western blot analysis of single clones transfected with *PS2* sgRNA and Cas9 proteins as shown in Extended Data Fig. 2-1). One allele was completely deleted (lower), while another allele was inverted between two sgRNA sites (upper).

**Figure 3.**
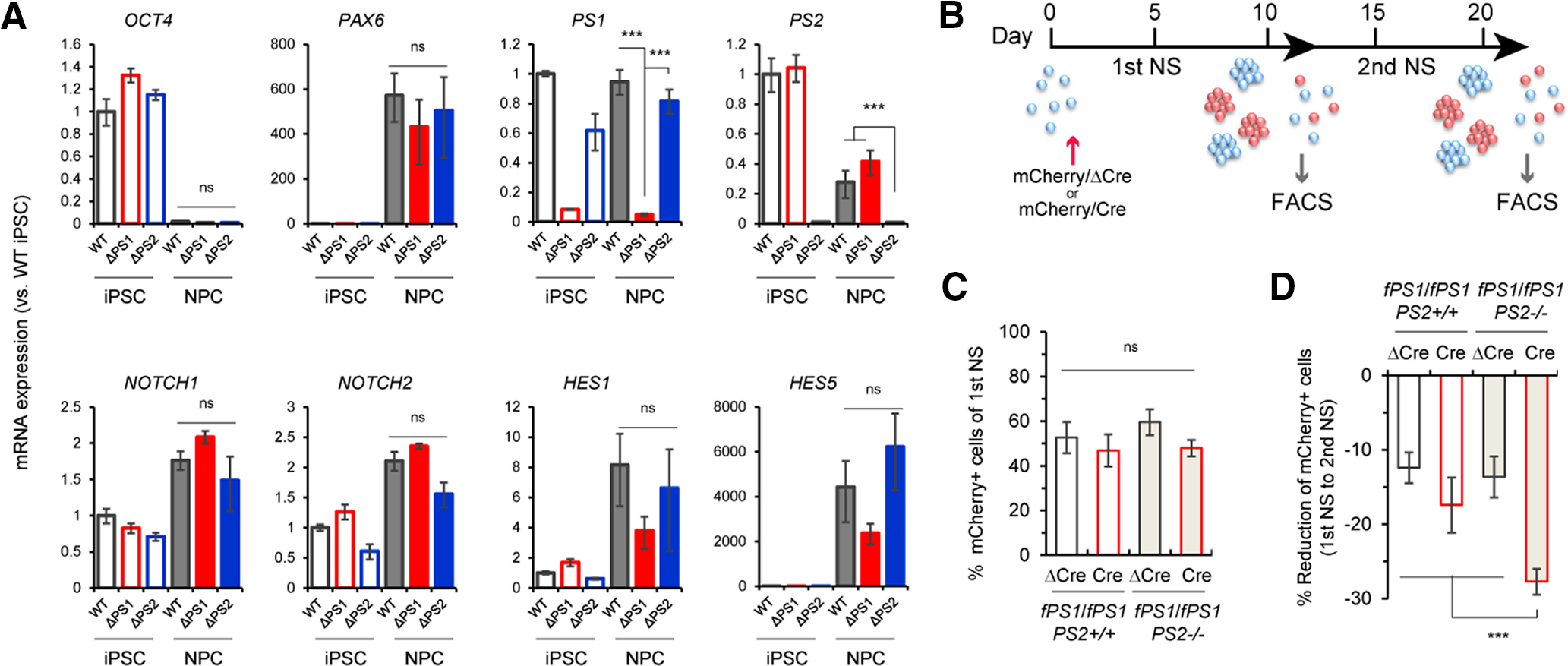
Maintenance deficit in iPSC-derived NPCs lacking PS1 and PS2. ***A***, qRT-PCR analysis of iPSCs and NPCs with the genotypes *fPS1/fPS1*;*PS2*^+/+^ (WT), *PS1*^−/−^;*PS2*^+/+^ (ΔPS1), and *fPS1/fPS1*;*PS2*^−/−^ (ΔPS2). Quantitative analysis shows the almost complete elimination of *PS1* and *PS2* in the ΔPS1-derived and ΔPS2-derived cells, respectively. Specific genes encoding components of the Notch signaling pathway (*NOTCH1*, *NOTCH2*, *HES1*, and *HES5*) were robustly increased in NPCs throughout neural patterning. The levels of both *HES1* and *HES5* were slightly but not significantly decreased in ΔPS1-NPCs compared with those in WT NPCs, whereas the levels of *NOTCH1* and *NOTCH2* were comparable among the genotypes. Data represent the mean ± SEM (*n* = 3–5 of independent culture batches). ns, not significant; ***p <* *0.001 by Tukey’s test among the three genotypes in the NPC group. ***B***, Experimental scheme of the colony-forming assay using *fPS1*/*fPS1*;*PS2*^+/+^ and *fPS1*/*fPS1*;*PS2*^−/−^ iPSCs by flow cytometry. ***C***, Quantitative analysis of the first neurospheres showing mCherry fluorescence. The fluorescence percentage was measured by flow cytometry after “partial” infection with lentivirus expressing *mCherry-ΔCre* or *mCherry-Cre* in *fPS1*/*fPS1*;*PS2*^+/+^ and *fPS1*/*fPS1*;*PS2*^−/−^ neurospheres (NS; flow cytometry plots were shown in Extended Data Fig. 3-1). Data represent the mean ± SD (*n *=* *3 assays). ns, not significant by Tukey’s test among the genotypes. ***D***, Quantitative analysis of the second neurospheres showing mCherry fluorescence. The fluorescence percentage was measured by flow cytometry and then calculated as the % reduction in mCherry^+^ cells from the first NS to the second NS (flow cytometry plots were shown in Extended Data Fig. 3-1). Data represent the mean ± SD (*n *=* *3 assays); ***p <* *0.001 by Tukey’s test among the genotypes.

**Figure 4.**
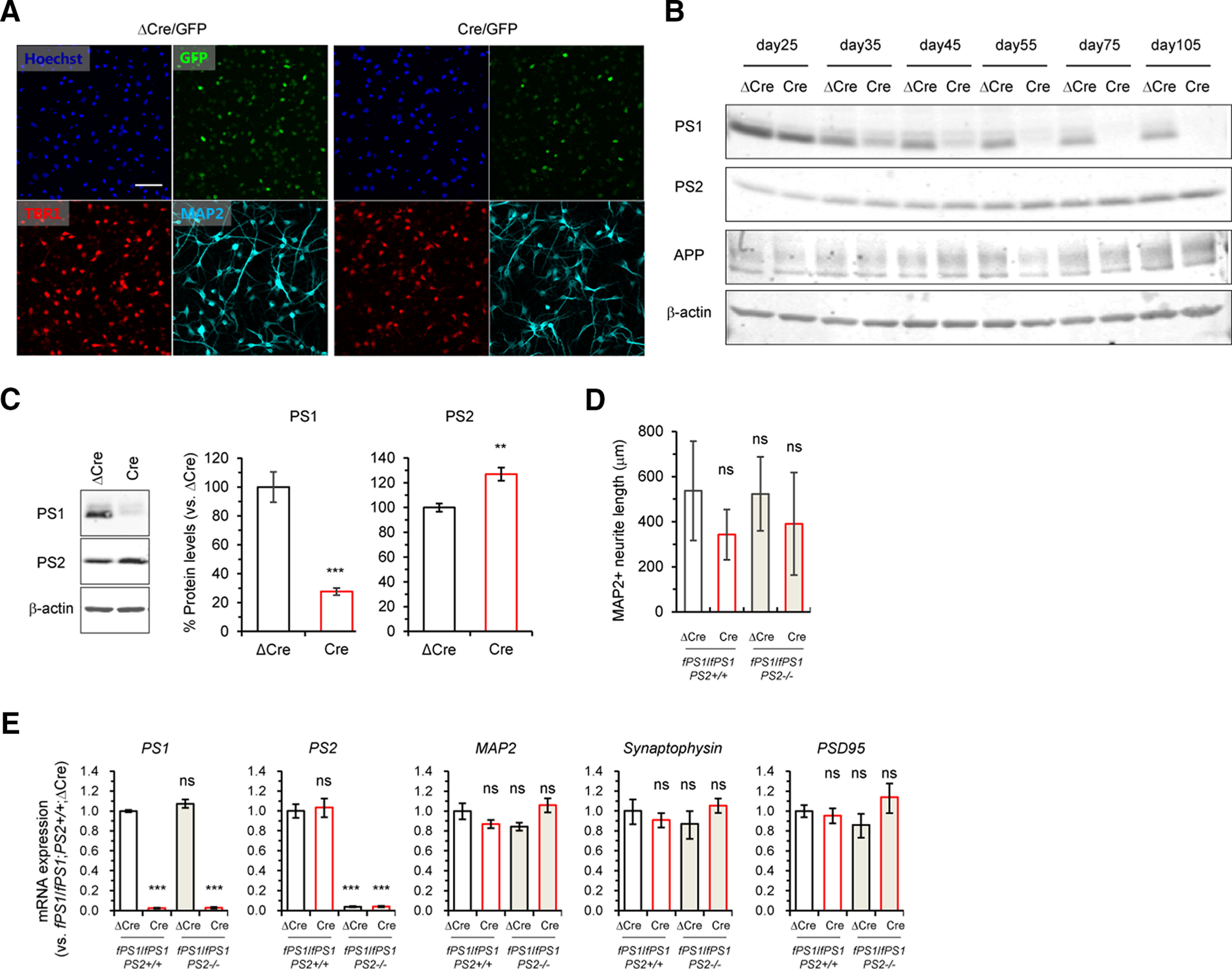
PS1/PS2 expression in genome-edited iPSCs harboring floxed *PS1* alleles following *Cre* expression. ***A***, Representative images of iPSC neurons (day 45) stained with antibodies specific for TBR1 and MAP2 are shown. Nuclear-localized GFP signals indicate iPSC neurons successfully infected with lentiviruses expressing *EGFP-ΔCre* or *EGFP-Cre*. Scale bar: 50 μm. ***B***, Western blot analysis of PS1-NTF, PS2-CTF, APP, and β-actin throughout terminal neuronal differentiation. Lentiviruses were infected at day 20. Because of posttranslational modification such as glycosylation, full-length of APP proteins exhibits two bands ∼100 kDa in size. ***C***, Western blot analysis of PS1-NTF, PS2-CTF, and β-actin. Representative blots are shown for each protein in *fPS1*/*fPS1* iPSC neurons infected with ΔCre or Cre lentivirus. Data represent the mean ± SEM (*n *=* *3 of independent culture batches); **p <* *0.01, ***p <* *0.001 by Student’s *t* test between the genotypes. ***D***, Quantification of MAP2+ neurite length at 45 d in *fPS1*/*fPS1*;*PS2*^+/+^ and *fPS1*/*fPS1*;*PS2*^−/−^ iPSC neurons with infected with ΔCre or Cre lentivirus showed no alteration among the genotypes, despite a slight shorter tendency in *PS1*-deficient neurons. Data represent the mean ± SD (*n* = 5 of independent culture batches). ns, not significant by Dunnett’s test versus the control. ***E***, qRT-PCR analysis at 45 d in *fPS1*/*fPS1*;*PS2*^+/+^ and *fPS1*/*fPS1*;*PS2*^−/−^ iPSC neurons infected with ΔCre or Cre lentivirus showed the almost complete elimination of *PS1* and *PS2* in *Cre*-infected neurons and *PS2*^−/−^ neurons, respectively, whereas the levels of neuronal markers such as *MAP2*, *synaptophysin*, and *PSD95* were comparable among the genotypic groups. Data represent the mean ± SEM (*n* = 3–4 of independent culture batches). ns, not significant; ***p <* *0.001 by Dunnett’s test versus the control.

**Figure 5.**
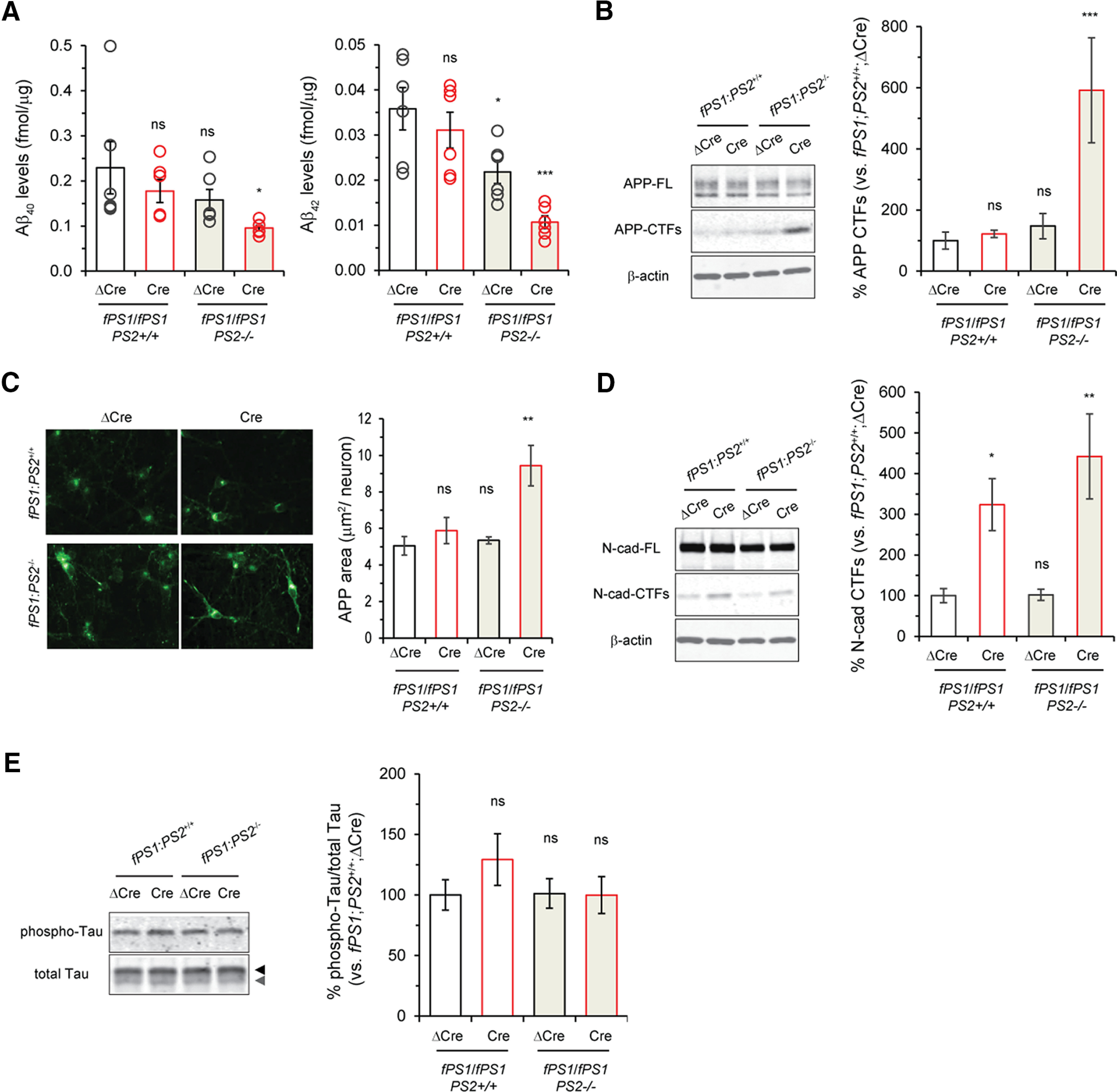
Cleavage of APP and N-cadherin in the iPSC neurons of *fPS1*/*fPS1*;*PS2*^+/+^ and *fPS1*/*fPS1*;*PS2*^−/−^ infected with ΔCre or Cre lentivirus. ***A***, Specific ELISA measurement of Aβ40 and Aβ42 in the iPSC neurons of *fPS1*/*fPS1*;*PS2*^+/+^ and *fPS1*/*fPS1*;*PS2*^−/−^ infected with ΔCre or Cre lentivirus. Quantitative analysis revealed a reduction in the Aβ levels in the iPSC neurons of *fPS1*/*fPS1*;*PS2*^−/−^ infected with *Cre* lentivirus. Data represent the mean ± SEM (*n* = 4–6 of independent culture batches); *p < 0.05, ***p < 0.001 by Dunnett’s test versus the control. No change of Aβ generation in the absence of PS1 alone was corroborated with the iPSC-neurons derived from another *fPS1*/*fPS1*;*PS2*^+/+^ clone (#249–3; Extended Data Fig. 5-1*A*). DAPT treatment nearly abolished the generation of Aβ40 and Aβ42 (Extended Data Fig. 5-1*C*). ***B***, Levels of APP-CTFs were quantified by Western blotting. Quantification analysis shows a massive increase in APP-CTFs in the iPSC neurons of *fPS1*/*fPS1*;*PS2*^−/−^ infected with *Cre* lentivirus. No accumulation of APP-CTFs in human neurons devoid of either *PS1* or *PS2* alone was further corroborated with the iPSC neurons from WT, ΔPS1, and ΔPS2 (Extended Data Fig. 5-1*D*). Data represent the mean ± SEM (*n* = 4–5 of independent culture batches). ns, not significant; ***p < 0.001 by Dunnett’s test versus the control. ***C***, Levels of the APP-CTFs were quantified by immunocytochemistry. Quantification analysis showed a massive increase of the APP-CTFs in the iPSC-neurons of *fPS1*/*fPS1*;*PS2*^−/−^ infected with *Cre* lentivirus. Data represent the mean ± SEM (*n* = 4–6 of independent culture batches). ns, not significant; **p < 0.01 by Dunnett’s test versus the control. ***D***, Levels of N-cadherin-CTF1s were quantified by Western blotting. In contrast to APP cleavage, N-cadherin was cleaved exclusively by PS1/γ-secretase. Data represent the mean ± SEM (*n* = 4–5 of independent culture batches). ns, not significant; *p < 0.05, ***p < 0.001 by Dunnett’s test versus the control. ***E***, Levels of phosphorylated (AT8) and total tau (Dako) were quantified by Western blotting. Most tau proteins in iPSC-derived neurons were phosphorylated (black arrowhead) rather than unphosphorylated form (gray arrowhead) using total tau antibody, similar to embryonic brains. The state of tau phosphorylation was not altered by the absence of PS1 and/or PS2. Data represent the mean ± SEM (*n* = 3–4 of independent culture batches). ns, not significant by Dunnett’s test versus the control.

**Figure 6.**
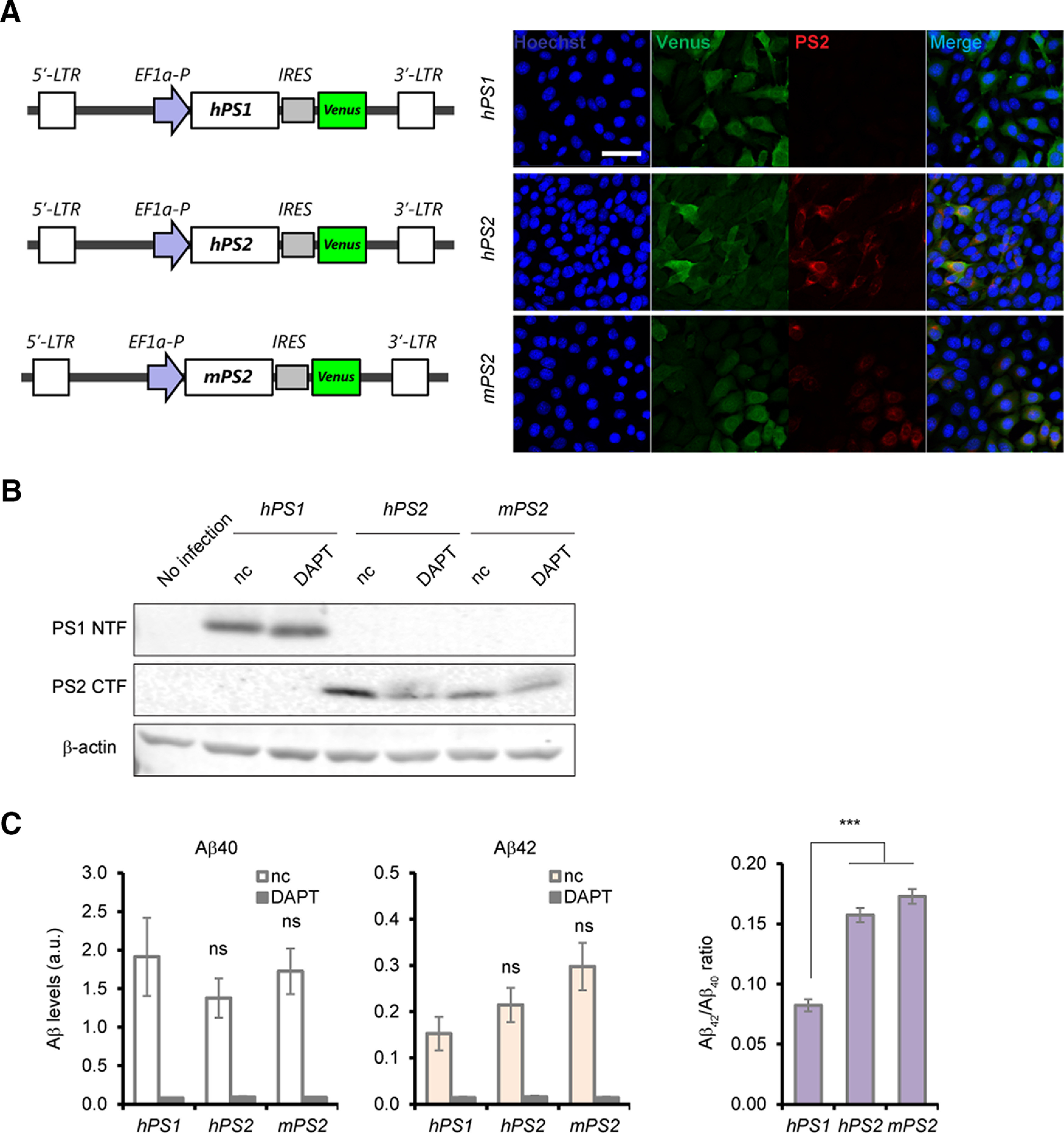
Comparable levels of Aβ secretion in mouse embryonic fibroblast cultures expressing human or mouse *PS2* heterologously. ***A***, Schematic diagram of lentivirus expressing human *PS1*, human *PS2* or mouse *PS2*, along with *Venus*. Representative pictures of immunocytochemistry in lentivirus-infected cultures. ***B***, Expression of PS1 and PS2 was analyzed in cell lysates of DKONL cultures expressing the construct by Western blotting. nc, DMSO (0.01%) treatment. ***C***, ELISA measurement specific for Aβ40 and Aβ42 in the DKONL cultures expressing hPS1, hPS2, or mPS2. The levels of Aβ were normalized with protein concentration and Venus protein level of the cell lysate. Data represent the mean ± SEM (*n* = 4 of independent culture batches). Calculated Aβ42/Aβ40 ratio was also drawn from the data measured by specific ELISA. ns, not significant; ***p <* *0.005 by Tukey’s test among the genotypes.

**Figure 7.**
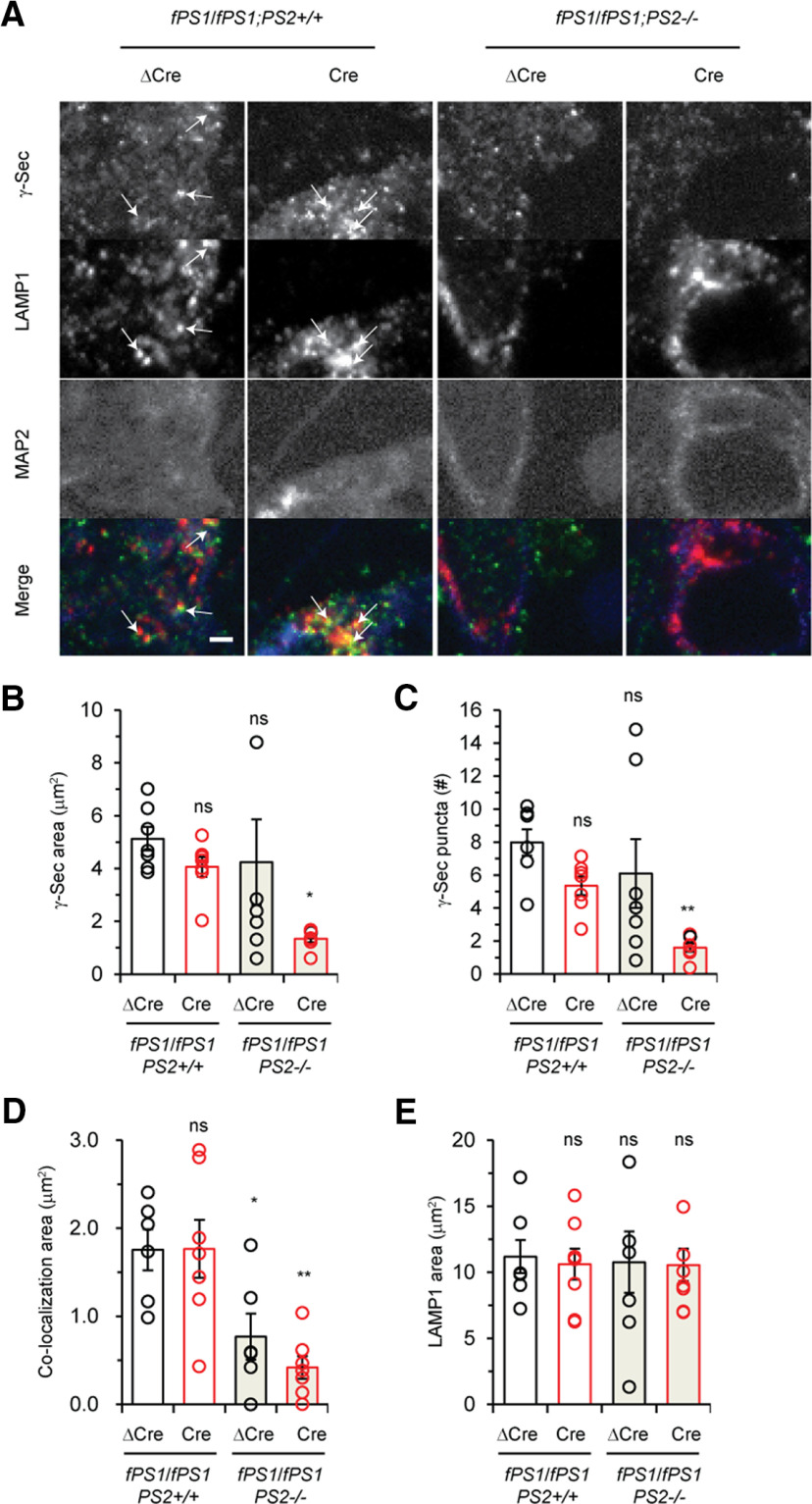
Subcellular localization of PS1/γ-secretase complexes and PS2/γ-secretase complexes. ***A***, Representative images of iPSC neurons stained with antibodies specific for γ-secretase complexes (A5226A), LAMP1 and MAP2 are shown. There are many puncta of γ-secretase complexes and LAMP1 in the perinuclear region and neurites. Arrows indicate colocalization puncta. Scale bar: 2 μm. The similar experiments using EEA1 antibody were shown in Extended Data Figure 7-1. ***B***, ***C***, Quantification of the γ-secretase complex puncta area (***B***) or number (***C***) per lentivirus-infected neuron. Data represent the mean ± SEM (*n* = 5–7 of independent culture batches). ns, not significant; *p < 0.05, **p < 0.01 by Dunnett’s test versus the control. ***D***, Colocalization of γ-secretase complexes and LAMP1. Quantification of these signals shows a decrease in LAMP1 colocalization in the absence of PS2. Data represent the mean ± SEM (*n* = 5–7 of independent culture batches). ns, not significant; *p < 0.05, **p < 0.01 by Dunnett’s test versus the control. ***E***, Quantification of the LAMP1 puncta area in lentivirus-infected neurons. Data represent the mean ± SEM (*n* = 5–7 of independent culture batches). ns, not significant by Dunnett’s test versus the control.

Data are presented as the mean value ± SEM, except Figures 3*C*,*D*, 4*D* (mean ± SD). The data in Figures 1–7, Extended Data Figures 5-1, 7-1 were analyzed using one-way analysis of variance and *post hoc* Dunnett’s or Tukey’s test. The data in Figure 4*C* and Extended Data Figure 5-1*A* were analyzed using Student’s *t* test. Statistical significance was defined as *p* < 0.05. The data were analyzed using R version 4.0.0 (The R Foundation).

### Ethics approval and consent to participate

Human ethics approval was obtained from the Ethics Committee in Keio University School of Medicine (approval number 20080016).

## Results

### Expression profile of each γ-secretase subunit in human iPSC-derived cortical neurons

γ-Secretase is widely expressed in nearly all cell types of multicellular organisms. However, the expression of every subunit of γ-secretase in human neural development remains to be determined, whereas many studies have focused Aβ production in human iPSC-derived neurons (Yagi et al., 2011; Yahata et al., 2011; Israel et al., 2012; Sho et al., 2020). We first examined how γ-secretase subunits are expressed throughout human neural differentiation using the efficient and robust differentiation protocol of iPSC-derived cortical neurons (Fig. 1*A*). The human neurons derived from a healthy control iPSC line exhibited characteristics of mature forebrain cortical neurons with βIII-tubulin-positive intricate neurites, and over 80% of them were positive for NeuN and TBR1 at day 45 (Fig. 1*B*). Next, to investigate the expression of each γ-secretase subunit during neural differentiation, we performed qRT-PCR using iPSCs, NPCs at day 14 and well-developed cortical neurons at day 45 (Fig. 1*C*). Both catalytic homologs, *PS1* and *PS2*, were gradually expressed in the course of differentiation and showed the highest expression in cortical neurons. Intriguingly, *PS2* mRNAs were drastically upregulated in neurons by ∼4-fold compared with those in iPSCs and NPCs, suggesting that PS2 plays important roles in mature human neurons. Among other essential subunits, *Aph-1a* and *Pen-2* were specifically increased in cortical neurons, whereas *nicastrin* and *Aph-1b* were expressed at comparable levels during differentiation. *APP* mRNAs were also upregulated in cortical neurons compared with those in iPSCs and NPCs, indicating that APP function is more important in mature neurons such as a putative neuronal adhesive protein (Sosa et al., 2017). More strikingly, expression of *BACE1*, a critical and rate-limiting enzyme for Aβ production, was significantly increased by ∼20-fold in neurons compared with that in iPSCs. These results suggest that iPSC-derived mature neurons are the most important source of Aβ rather than iPSCs and NPCs.

### Generation of human stem cell models for assessing γ-secretase physiological functions

γ-Secretase is essential for embryonic development exclusively via regulation of the Notch pathway (De Strooper et al., 1999). Indeed, the deficiency of crucial components of γ-secretase such as the *PS1*, *Aph-1a*, or *Pen-2* genes causes embryonic or perinatal lethality in mice (Shen et al., 1997; Wong et al., 1997; Ma et al., 2005; Bammens et al., 2011), which phenocopies *Notch1*-null mice (Swiatek et al., 1994). As the Notch pathway is important for the generation and maintenance of NPCs (Handler et al., 2000; Kim and Shen, 2008), simple KO of PS1 could cause a deleterious disturbance in neural differentiation from PSCs. We thus used a cKO method using a Cre/*loxP* system to uncover the normal functions of γ-secretase in iPSC-derived neural lineages. More importantly, this state-of-the-art strategy can circumvent the frequent problems caused by iPSC clonal variability as well (Cahan and Daley, 2013; Liang and Zhang, 2013).

The *PS1* targeting vector includes the 5′ homologous region (∼2.3 kb) containing exons 2–3, a selection cassette (PGK-puroΔtk or PGK-neoΔtk) flanked by *FRT* sequences and the 3′ homologous region (∼1.1 kb), in which exons 2–3 are adjacent to two *loxP* sites in the 5′- and 3′- homologous regions (Fig. 2*A*). Homozygous targeted *PS1* iPSCs, in which the proper homologous recombination events occurred successfully, were confirmed by sequencing. Because PS1 is expressed in iPSCs, we first examined genome-edited iPSCs to determine whether the floxed exons can be recombined and whether expression of the PS1 protein is actually abrogated. Genomic DNAs from homozygous targeted *PS1* iPSCs transfected with a plasmid expressing *FLP* or *Cre* were analyzed with a primer pair encompassing the *loxP* sites and the selection cassette. FLP and Cre can delete a region flanked by *FRT* and *loxP* sequences, respectively (Fig. 2*B*). We next performed Western blotting to investigate whether removal of exons 2–3 leads to ablation of PS1 proteins. PS1-NTFs and PS1-CTFs were almost completely eliminated in the iPSCs carrying homozygous deleted alleles, whereas the expression levels were comparable among parental cell (201B7) and iPSCs harboring the targeted or floxed alleles (Fig. 2*C*). These results clearly demonstrated that this *PS1* cKO system works correctly on *Cre* introduction, by which PS1 proteins are eliminated. In the following experiments, we used iPSCs harboring homozygous floxed *PS1* alleles, where removal of the selection cassette from targeted alleles occurred successfully.

10.1523/ENEURO.0500-20.2021.f2-1 Extended Data Figure 2-1 PS2 Western blot analysis of single clones transfected with *PS2* sgRNA and Cas9 proteins. Two clones #10 and #12 were nearly negative for PS2 proteins. Download Figure 2-1, TIF file.

It has been reported that PS2, another homolog of presenilin in vertebrates, can compensate for a lack of PS1 in mice (Lai et al., 2003; Watanabe et al., 2014). To eliminate this compensation by PS2, we further deleted exon 5 in the *PS2* gene by using the CRISPR/Cas9 system (Fig. 2*D*). Out of 12 clones derived from *fPS1*/*fPS1* iPSCs, we screened 2 clones lacking PS2 protein expression (Extended Data Fig. 2-1) and confirmed the genomic sequence around exon 5, which could not produce the inherent PS2 mRNAs and/or proteins (Fig. 2*E*). Finally, we obtained two isogenic human iPSCs bearing genome-edited *PS* genes: *fPS1*/*fPS1*;*PS2^+/+^* and *fPS1*/*fPS1*;*PS2*^−/−^.

### Deleterious effects of presenilin deficiency on Notch-dependent NPC maintenance in *PS*-deficient iPSC-derived neural stem cells

Loss of PS1 or PS1/PS2 results in the depletion of neural stem cells in mice, as the Notch signaling pathway is severely compromised (Handler et al., 2000; Hitoshi et al., 2002; Kim and Shen, 2008). To examine the effect of *PS* deficiency on Notch signaling, we first induced the differentiation of *fPS1*/*fPS1*;*PS2^+/+^* (WT), *PS1*^−/−^;*PS2^+/+^* (*ΔPS1*), where the *PS1* gene was already KO out by Cre, and *fPS1*/*fPS1*;*PS2*^−/−^ (*ΔPS2*) iPSCs into NPCs at day 14 and performed qRT-PCR to examine several genes related to the Notch pathway (Fig. 3*A*). *OCT4* mRNAs were nearly absent in NPCs, whereas expression of *PAX6*, a forebrain NPC marker, was induced, suggesting a successful escape from the pluripotency state. As expected, *PS1* and *PS2* mRNAs were almost completely eliminated in iPSCs/NPCs derived from *ΔPS1* and *ΔPS2* iPSCs, respectively. Both *NOTCH1* and *NOTCH2* expression were increased significantly in NPCs compared with those in iPSCs without any genotypic effects. Interestingly, the Notch target genes *HES1* and *HES5* were also robustly upregulated in NPCs, especially *HES5,* which was upregulated by ∼5000-fold, indicating that in iPSC-derived human NPCs, HES5 is a main downstream effector of Notch signaling. Surprisingly, the expression of *HES1* and *HES5* was slightly but not significantly decreased in *ΔPS1* NPCs compared with those in WT and *ΔPS2* NPCs (*p *=* *0.225 for *HES1* and *p *=* *0.185 for *HES5* compared with the WT by Dunnett’s test). The inefficacious effect of *PS1* deficiency on *HES5* reduction in human NPCs is inconsistent with the results in prior literature showing a significant decrease in *Hes5* in the telencephalon and anterior diencephalon in *PS1*-null mouse (Handler et al., 2000). These results suggest that PS2/γ-secretase-mediated Notch activity can sufficiently compensate for the lack of PS1 in human NPCs.

10.1523/ENEURO.0500-20.2021.f3-1 Extended Data Figure 3-1 Flow cytometry analysis in colony forming assay. Dissociated neurospheres were fractionated by single cell gate and analyzed with mCherry fluorescence. The representative histograms were shown from both primary and secondary NS analyses. Note that mCherry fluorescence got stronger in secondary NS due to a delayed expression from lentivirus in primary NS. Download Figure 3-1, TIF file.

As the loss of PS1 alone in NPCs tended to affect Notch signaling negatively, albeit not significantly, we further investigated the effects of PS deficiency on Notch-mediated neural stem cell potency. To avoid any possible disadvantages for iPSCs caused by the long-term absence of PS, we applied a clonal colony-forming assay using *fPS1*/*fPS1*;*PS2^+/+^* and *fPS1*/*fPS1*;*PS2*^−/−^ iPSCs, by which PSCs can be initially induced in serum-free medium to generate neurospheres (Chaddah et al., 2012; Imaizumi et al., 2015, 2018; Fujimori et al., 2017). To investigate whether a lack of PS causes any effects in iPSC-derived neurospheres, lentivirus expressing *mCherry-Cre* or *mCherry-ΔCre* was used for infection at a MOI ≈1 at the very beginning of the first neurosphere formation (Fig. 3*B*). We then measured the mCherry^+^ cell population by flow cytometry following dissociation at the end of the first and second neurosphere formation (Fig. 3*C*,*D*; Extended Data Fig. 3-1). Intriguingly, mCherry^+^ cells in the second neurosphere of *fPS1*/*fPS1*;*PS2*^−/−^;*Cre* (20.3 ± 2.0%) were significantly decreased by ∼30% compared with those in the first neurosphere (48.0 ± 3.7%), whereas *ΔCre*-infected cells showed a slight decrease between the first (59.6 ± 5.8%) and second (45.9 ± 8.0%) neurospheres. In contrast, *fPS1*/*fPS1*;*PS^+/+^*;*Cre* showed a tendency toward the reduction of the mCherry^+^ population by ∼17% [*p *=* *0.055 according to Student’s *t* test between the first neurosphere (46.9 ± 7.2%) and second neurosphere (29.5 ± 8.7%)], which is consistent with the qRT-PCR results of Notch signaling, as shown in Figure 3*A*. Together, these results demonstrate that the loss of both PS1 and PS2 impairs the growth and/or maintenance of human neurospheres.

### Minimal developmental effect of PS inactivation on mature human neurons

To investigate whether the absence of PS1, PS2, or both affects neuronal differentiation in iPSC-derived cortical neurons, we first developed a cKO cortical neuronal culture system, in which PS1 can be eliminated by *Cre* transduction at any time. To circumvent any possible effects of *PS* deficiency during neural progenitor maintenance (Fig. 3), we infected *fPS1*/*fPS1*;*PS2^+/+^* and *fPS1*/*fPS1*;*PS2*^−/−^ cells with lentivirus expressing *Cre* 5–6 d after terminal differentiation (day 20). When infected at a MOI = 3, ∼90% of cells were positive for fluorescence in cortical neurons at day 45 (Fig. 4*A*). To determine exactly when PS1 was eliminated in this system, we performed Western blot analysis using *fPS1*/*fPS1*;*PS2^+/+^* neuronal cell lysates at 5–85 d after infection with the lentivirus expressing *ΔCre* or *Cre*. The levels of PS1 proteins were robustly decreased at 15 d after *Cre* transduction (day 35), and the extent of reduction progressively increased (Fig. 4*B*), whereas the levels of APP protein were relatively comparable. Intriguingly, the level of PS1 and PS2 proteins was gradually changed in *ΔCre*-infected cultures during neuronal maturation period (day 25–105) regardless of insufficient trials (Fig. 4*B*), consistent with prior report showing that PS is expressed differentially during mouse brain development (Kumar and Thakur, 2012). We quantified PS protein levels 25 d after *Cre* transduction (day 45) and found that the PS1 level was significantly reduced by ∼80% compared with that in *ΔCre*-infected cultures (Fig. 4*C*). Interestingly, the PS2 level was significantly increased by ∼25% in *Cre*-infected cultures, suggesting the presence of compensation for PS1 deficiency. To investigate whether the loss of PS affects neuronal differentiation in the cultures, we next performed immunocytochemistry using MAP2 antibody and measured neurite length of *fPS1*/*fPS1*;*PS2^+/+^*;*ΔCre* (control), *fPS1*/*fPS1*;*PS2^+/+^*;*Cre* (*PS1*-null), *fPS1*/*fPS1*;*PS2*^−/−^;*ΔCre* (*PS2*-null) and *fPS1*/*fPS1*;*PS2*^−/−^;*Cre* (*PS1*/*PS2*-null) neurons at day 45, in which lentiviruses were infected 5 d after terminal differentiation. Neurite length was almost comparable among the genotypes, despite a slight decrease in *Cre*-infected neurons (Fig. 4*D*), indicating negligible effect of *PS* absence on neuronal morphology in fully differentiated neurons. We further performed qRT-PCR of several neuronal markers. *PS1* and *PS2* mRNAs were almost absent in the neuronal cultures devoid of PS1 (*PS1*-null and *PS1*/*PS2*-null) and PS2 (*PS2*-null and *PS1*/*PS2*-null), respectively, whereas the levels of all neuronal markers, including *MAP2*, *synaptophysin*, and *PSD95*, were comparable among the genotypes (Fig. 4*D*). Together, these results demonstrated that specific elimination of PS can be accomplished without any gross defects in neuronal differentiation.

### Disturbed APP processing in *PS*-deficient human neurons

Aβ peptides are generated by sequential cleavage of the APP protein by β-secretase and γ-secretase (De Strooper et al., 1998; Vassar et al., 1999). To investigate how the elimination of PS1, PS2 or both affects Aβ production in iPSC-derived cortical neurons, we first performed sandwich ELISAs specific for Aβ40 and Aβ42 using culture medium from neuronal cultures at day 45. Surprisingly, the levels of Aβ40 were decreased only in *PS1*/*PS2*-null neuronal cultures, whereas cultures with other genotypes showed comparable levels of Aβ40 (Fig. 5*A*). Another independent iPSC line harboring *fPS1*/*fPS1*;*PS2^+/+^* also exhibited no decrease in Aβ in the absence of PS1 alone (Extended Data Fig. 5-1*A*), excluding the possibility of clonal variability. Furthermore, the levels of Aβ42 were decreased in both *PS2*-null and *PS1*/*PS2*-null neuronal cultures but not in *PS1*-null cultures, suggesting the importance of PS2/γ-secretase in Aβ42 production (Fig. 5*A*). Notably, the ratio of Aβ42/Aβ40 was not increased in *PS1*-null and/or *PS2*-null neuronal cultures (Extended Data Fig. 5-1*B*), unlike the case of FAD-linked *PS1* mutations (Yagi et al., 2011; Woodruff et al., 2013; Kondo et al., 2017; Ishikawa et al., 2020; Sho et al., 2020). Inhibition of γ-secretase with DAPT abolished the secretion of Aβ40 and Aβ42 (Extended Data Fig. 5-1*C*), indicating the successful measurement of γ-secretase-mediated Aβ production; however, low levels of Aβ were generated even with DAPT treatment, which is supposedly an APP cleavage product by a γ-secretase-independent proteolysis (Ladror et al., 1994; Cataldo et al., 1995). These results demonstrated that inactivation of PS1 alone is not enough to eliminate Aβ production in human cortical neurons, which is inconsistent with previous reports showing that Aβ40 and Aβ42 are significantly reduced in *PS1* cKO mice (Yu et al., 2001; Watanabe et al., 2014). We further examined another index of APP processing in *PS*-deficient human neurons. Aβ peptides were produced directly from APP-CTFs, which are products cleaved by β-secretase and are the direct substrate of γ-secretase. Therefore, the accumulation of APP-CTFs is a good indicator of γ-secretase impairment. We performed Western blotting using an antibody for the APP C-terminal region and found that APP-CTFs were accumulated only in the lysates from *PS1*/*PS2*-null neuronal cultures (Fig. 5*B*), which is consistent with the results of the Aβ ELISAs. We further performed immunocytochemistry of APP-CTFs, and again, the signals were robustly increased only in *PS1*/*PS2*-null neurons (Fig. 5*C*). These results prompted us to test whether PS1 elimination is effective enough in producing subsequent phenotypes in *PS1*-null neurons. When we used cortical neurons derived from *PS1* KO (*ΔPS1*) iPSCs in which the *PS1* gene was already knocked out, an accumulation of APP-CTFs was not observed (Extended Data Fig. 5-1*D*), demonstrating that a lack of PS1 alone is not enough to compromise APP processing. Next, we examined another substrate of γ-secretase, N-cadherin, which is expressed in mature neurons as an essential hemophilic adhesion molecule at synapses (Fannon and Colman, 1996; Uchida et al., 1996). N-cadherin is subjected to sequential cleavage by ADAM10 and γ-secretase (Marambaud et al., 2003; Reiss et al., 2005; Uemura et al., 2006). Ncad-CTF1, a cleaved product of N-cadherin produced by ADAM10, was significantly accumulated in both *PS1*-null and *PS1*/*PS2*-null neurons according to Western blot analysis (Fig. 5*D*). These results revealed that *Cre*-mediated PS1 elimination in this system is sufficient to assess the effects on substrate processing in human cortical neurons. Together, our novel human neuronal system clarified the substrate specificity of PS1 and PS2. Considering that Aβ has a causative relation with other pathologic lesions in the course of AD pathogenesis, we next examined whether acute ablation of PS1 and/or PS2 affects phosphorylation state of tau, another pathologic hallmark in AD patient’s neurons. Unexpectedly, tau phosphorylation was not increased in *PS*-deficient neurons compared with *PS* intact neurons, indicating that tau phosphorylation is not directly regulated by PS/γ-secretase (Fig. 5*E*).

10.1523/ENEURO.0500-20.2021.f5-1 Extended Data Figure 5-1 Processing of APP in the *PS*-null iPSC-derived neurons. ***A***, ELISA measurement specific for Aβ40 and Aβ42 in the iPSC-neurons from another *fPS1*/*fPS1*;*PS2*^+/+^ clone (#249-3) infected with ΔCre or Cre lentivirus. Data represent the mean ± SEM (*n *=* *3 of independent culture batches). ns, not significant by Student’s *t* test between the genotypes. ***B*,** Calculated Aβ42/Aβ40 ratio was also drawn from the data in Figure 5*A*. Data represent the mean ± SEM (*n *=* *3 of independent culture batches). ns, not significant; *p < 0.05 by Dunnett’s test versus the control. ***C***, ELISA measurement specific for Aβ40 and Aβ42 in the iPSC-neurons of *fPS1*/*fPS1*;*PS2*^+/+^ and *fPS1*/*fPS1*;*PS2*^-/-^ infected with ΔCre or Cre lentivirus, with DAPT treatment for 48 h. Data represent the mean ± SEM (*n* = 4–5 of independent culture batches). ns, not significant by Dunnett’s test versus the control. ***D***, Levels of the APP-CTFs are quantified in WT, ΔPS1, and ΔPS2 neurons by Western blotting. No alteration of APP-CTFs was observed among the genotypes. Data represent the mean ± SEM (*n* = 3–4 of independent culture batches). ns, not significant by Dunnett’s test versus the control. Download Figure 5-1, TIF file.

As several amino acid residues are different between the human and mouse PS2 protein (Levy-Lahad et al., 1995; Rogaev et al., 1995; Vito et al., 1996), we first hypothesized the critical differences in PS2/γ-secretase activity per se between humans and mice, which are attributed to inconsistent APP cleavage in *PS1*-deficient cells. To elucidate whether human and mouse PS2/γ-secretase exhibit specific APP processing in the same *PS*-deficient cellular context, we performed a PS2 complementation assay using *PS*-deficient mouse fibroblast cells, which stably express Swedish mutant APP (Fig. 6*A*,*B*). When we expressed human PS1 exogenously using an *EF1α* promoter-driven lentivirus along with an IRES-mediated Venus fluorescent protein (Nagai et al., 2002), Aβ peptides were robustly generated in the culture medium, in which DAPT almost completely abolished Aβ secretion (Fig. 6*C*). We next expressed human and mouse PS2 and measured Aβ. PS2 from both species could secrete Aβ peptides at equivalent levels when normalized to the expression levels of the Venus protein (Fig. 6*C*). Interestingly, we found that PS2 significantly increased the Aβ42/Aβ40 ratio compared with PS1, although there was no large difference in Aβ secretion between PS1 and PS2. Using this exogenous expression system, we demonstrated that PS2/γ-secretase itself does not exhibit species differences in its activity, and PS2-directed activity generated a longer form of Aβ compared with PS1-directed activity; the latter findings are consistent with those of the Aβ ELISAs in *PS*-deficient human neurons (Fig. 5*A*).

### Unique subcellular localization of distinct γ-secretase complexes containing PS1 or PS2 in human neurons

Previous reports showed differences in the subcellular localization of γ-secretase complexes depending on their distinct subunit composition in human nonneuronal cell lines and mouse primary neurons, where the fluorescent protein-tagged γ-secretase component was expressed heterologously (Meckler and Checler, 2016; Sannerud et al., 2016). To elucidate the subcellular localization of endogenous PS1 or PS2/γ-secretase complexes in human neurons, we used a monoclonal antibody specific for glycosylated nicastrin (A5226A), which resides only in the active γ-secretase complex (Hayashi et al., 2012). Only a small number of subunits are involved in the assembly of the γ-secretase complex (Thinakaran et al., 1997; Kimberly et al., 2002); therefore, this antibody is useful to distinguish the nicastrin subunit in the γ-secretase complex from free nicastrin. We first performed immunocytochemistry and found that both the areas and the number of puncta positive for the γ-secretase complex were significantly reduced in *PS1/PS2*-null neurons compared with those in control neurons (Fig. 7*A–C*), indicating that active γ-secretase complex does not form in the absence of PS. However, some signals were detected even in *PS1/PS2*-null neurons, albeit with a robust reduction in the number of puncta, suggesting that a small proportion of nicastrin monomer can be recognized or that the γ-secretase complex formed before *Cre*-lentivirus infection still remains in the *PS1/PS2*-null neurons. We next examined the colocalization of γ-secretase and intracellular organelles using antibodies against EEA1 and LAMP1 as early endosome and late endosome/lysosome markers, respectively. The percentages of γ-secretase complexes costained with EEA1 and LAMP1 were 4.3 ± 0.6% and 40.4 ± 8.5%, respectively (Fig. 7; Extended Data Fig. 7-1), demonstrating that approximately half of the γ-secretase complex exists in LAMP1+ organelles in human neurons. Interestingly, colocalization signals for γ-secretase and LAMP1 were significantly decreased by ∼60% and ∼80% in *PS2*-null and *PS1/PS2*-null neurons compared with those in controls, respectively (Fig. 7*D*), although the area of LAMP1+ organelles was comparable among the four genotypes (Fig. 7*E*). These results are consistent with a recent report that PS2 is mainly localized in late endosomes/lysosomes in nonneuronal cells and rodent neurons (Meckler and Checler, 2016; Sannerud et al., 2016). Together, these results strongly suggest that PS2/γ-secretase localizes largely in LAMP+ organelles and that almost no γ-secretase stays in EEA1+ organelles in human neurons.

10.1523/ENEURO.0500-20.2021.f7-1 Extended Data Figure 7-1 No significant change of EEA1+ puncta in the iPSC-neurons of *fPS1*/*fPS1*;*PS2*^+/+^ and *fPS1*/*fPS1*;*PS2*^-/-^ infected with ΔCre or Cre lentivirus. ***A***, Representative images of iPSC-neurons stained with antibodies specific for γ-secretase complexes (A5226A), EEA1, and MAP2 are shown. There are many puncta of γ-secretase complexes and EEA1 in perinuclear region and neurites. Scale bar: 2 μm. ***B***, Quantification of EEA1+ puncta area in the iPSC-neurons, and no significant difference between the genotypes. Data represent the mean ± SEM (*n* = 4–6 of independent culture batches). ns, not significant by Dunnett’s test versus the control. ***C***, Size distribution of EEA1+ puncta of iPSC-derived neurons with the threshold of 1.0-μm^2^ puncta. Data represent the mean ± SEM (*n* = 4–6 of independent culture batches). ns, not significant by Dunnett’s test versus the control. Download Figure 7-1, TIF file.

Endosome enlargement is detected in AD patient brains and AD model-derived neurons as a characteristic cytopathology (Cataldo et al., 2000; Kwart et al., 2019). Because the impairment of proper APP processing and the concomitant accumulation of APP-CTFs are culprits for this phenomenon, we analyzed EEA1+ puncta in human neurons. Surprisingly, no enlargement in EEA1+ signals was observed in *PS1/PS2*-null neurons (Extended Data Fig. 7-1), despite the robust accumulation of APP-CTFs in these neurons (Fig. 5*B*,*C*). Therefore, it is most likely that a loss of PS causes an accumulation of APP-CTFs (Fig. 5*B*) but not an enlargement of early endosomes, which probably necessitates additional impairment, such as an APP/PS1 mutation, in addition to the accumulation of APP-CTFs (Kwart et al., 2019).

## Discussion

The use of gene KO methodology in animals or cultured cells is a conventional means to assess physiological and/or pathophysiological functions of the gene of interest. However, a simple KO strategy has sometimes been hampered by developmental disturbance: one such example is the *PS1* gene, the germline KO of which causes perinatal death in mice (Shen et al., 1997; Wong et al., 1997). To circumvent this developmental lethality in mice, both *PS1* cKO and *PS* cDKO mice have been created by crossing *floxed PS1* mice with *αCaMKII*-*Cre* transgenic mice, along with or without *PS2* KO mice, demonstrating that PS is essential for cortical neuron survival and synaptic functions in mice (Saura et al., 2004; Zhang et al., 2009). In this study, to investigate the physiological functions of PS in the human neural cell context, we developed a novel human iPSC-derived model in which PS1 is ablated with the Cre/*loxP* system to avoid any possible developmental impediment. Indeed, *PS1* cKO iPSCs can circumvent the maintenance deficit of *PS1/PS2*-null NPCs. By examining mature human neurons lacking PS, we clearly revealed the human-specific regulation of PS/γ-secretase and substrates (APP and N-cadherin) and compared them with previous murine-based results (Yu et al., 2001; Watanabe et al., 2014). The discrepancy may result from the inherent intracellular circumstances but not from primary sequence differences in the PS protein. Using a specific antibody against mature glycosylated nicastrin, we characterized the subcellular localization of nearly half of the endogenous PS/γ-secretase complex in late endosomes/lysosomes, where Aβ42 is relatively abundantly generated. These findings are the first to show the physiological function/location of an endogenous but not heterologously expressed PS/γ-secretase complex in a human neural cellular context.

γ-Secretase is composed of four integral subunits, PS, nicastrin, Aph-1, Pen-2 (Kimberly et al., 2003; Takasugi et al., 2003), and only one molecule from each subunit is assembled into the complex (Sato et al., 2007). Importantly, PS and Aph1 each have a homolog; PS1, PS2, Aph-1A (which further forms L and S variants by alternative splicing), and Aph-1B can form six putative γ-secretase complexes. Furthermore, γ-secretase cleaves many substrates, almost all of which are Type I transmembrane proteins such as APP and Notch, leading to nuclear signaling that results in transcription and proteostasis of membrane proteins (Haapasalo and Kovacs, 2011). One important unsettled question is whether distinct γ-secretase complexes can equally cleave their different target substrates. Previous reports have also revealed that there is relative substrate specificity among distinct γ-secretases in nonneuronal cell cultures and in mice (Serneels et al., 2009; Sannerud et al., 2016). In the present study using human neural cells, we demonstrated the exclusive specificity of endogenous PS1/γ-secretase toward N-cadherin, although such clear specificity has not been concluded in mouse fibroblasts (Marambaud et al., 2003). In this regard, it is most likely that PS1/γ-secretase performs N-cadherin cleavage strictly at the site lacking a PS2/γ-secretase in human neurons. However, it remains to be determined whether diverse γ-secretase complexes containing Aph-1A or Aph-1B show any substrate specificity in human neurons.

γ-Secretase and its substrates are both membrane proteins; thus, proteolytic reactions occur only in the same subcellular compartment. Indeed, several groups have demonstrated that the different subcellular localizations of each γ-secretase underlie their substrate specificity in nonneuronal cells (Tarassishin et al., 2004; Meckler and Checler, 2016; Sannerud et al., 2016). Sannerud and colleagues showed that PS2/γ-secretase is localized in late endosomes/lysosomes via the specific targeting signal of PS2 (Sannerud et al., 2016), which is consistent with our results in human neurons. However, in this study, a small proportion of γ-secretase still resided in LAMP1+ organelles in the absence of PS2. This may result from the compensatory expansion of PS1 in their location because of a loss of PS2. Alternatively, LAMP1 immunoreactivity is found more broadly in locations beyond the late endosome/lysosome, according to the recent literature (Cheng et al., 2018). In contrast, we could barely detect colocalization between EEA1+ organelles and γ-secretase, suggesting that EEA1+ early endosomes are a transient location of γ-secretase (Kanatsu et al., 2014). Furthermore, in relation to its exclusive N-cadherin cleavage (Fig. 5*D*), PS1/γ-secretase but not PS2/γ-secretase could be targeted in the plasma membrane, where ∼6% of total γ-secretase exists, as shown in cell lines (Chyung et al., 2005). Other membrane organelles, including the Golgi apparatus and recycling endosome, remain to be analyzed in the future.

In terms of APP processing, we and other groups have shown obvious species-specific or cell type-specific differences. Using *PS* KO cells, APP can be efficiently processed by both PS1 and PS2 in some systems (Lessard et al., 2019; Pimenova and Goate, 2020), including those examined in this study, whereas PS2 enzymatic activity is not efficient in others (Watanabe et al., 2014; Arber et al., 2019). This discrepancy might be a result of distinct PS2 expression levels in each system as well as differences in subcellular localization, as discussed above. Conceivably, this discrepancy could be partly caused by differences between neuronal and other neural cells. The elimination of PS1 in excitatory neurons of *PS1* cKO mice could affect other cell types, leading to non-cell autonomous effects on APP processing in neurons. Surprisingly, Woodruff et al., showed that human isogenic iPSCs carrying the PS1 ΔE9 mutation, which is a partial loss-of-function mutation, led to an accumulation of APP-CTFs despite the presence of a total of three WT *PS1*/*PS2* alleles (Woodruff et al., 2013). The difference in APP processing in iPSC-derived human neurons might result from the usage of different iPSC lines or neuronal differentiation protocols between the other study and ours. Alternatively, ΔE9-mutant PS1/γ-secretase could confer a putative dominant-negative effect on γ-secretase bearing WT PS1/PS2 (Watanabe and Shen, 2017).

Likewise, the scenario is similar for Notch processing. PS1 is crucial for neural development during embryogenesis through Notch signaling, and *PS1* KO homozygous mice exhibit a perinatal lethal phenotype (Shen et al., 1997; Wong et al., 1997). In this study, however, the extent of the PS1 contribution to the maintenance of the human neurosphere was much less than that of the mouse neurosphere (Hitoshi et al., 2002). This discrepancy might result from sufficient compensation by *PS2* in human *PS1*-deficient NPCs. Alternatively, as Notch pathway is modulated by other signaling pathways such as Sonic hedgehog, Bone morphogenetic proteins and Wnt (Gajera et al., 2010; Lin and Hankenson, 2011; Fujimori et al., 2017, 2018), we cannot exclude the possibility that other pathways could efficiently compensate the decreased Notch pathway in *PS1*-deficient NPCs. More interestingly, Arber and colleagues recently demonstrated that FAD-linked *PS1* mutations bring about precocious characters in iPSC-derived NPCs (Arber et al., 2021). Despite some discrepancy between their study (*PS1* mutation) and ours (*PS* deficiency), it is more likely that an impaired PS/γ-secretase resulted in disturbance of NPC maintenance. Collectively, these results clearly demonstrate the contextual differences between γ-secretase and its substrate, underscoring the importance of human neuronal models in the scrutiny of AD pathogenesis.

In this study, we clarified the heterogeneity of PS/γ-secretase complex in human cortical neurons, which underlies different production modes of endogenous Aβ species by individual PS/γ-secretase complex. Although our study would provide a unique cellular model to scrutinize the physiological production mechanism of specific Aβ species in the context of human neurons, this *PS* cKO iPSC model did not always mimic the authentic model of AD pathogenesis. Expectedly, the complete loss of PS function in human cortical neurons showed the negligible generation of both Aβ40 and Aβ42 (Fig. 5*A*), which results in an erratic decrease of Aβ42/Aβ40 ratio (Extended Data Fig. 5-1*B*). Given that most FAD-linked PS mutations cause a partial loss of γ-secretase function (Szaruga et al., 2015), especially carboxyl-peptidase activity, leading to an accumulation of toxic longer Aβ42 and Aβ43, the effect of FAD-linked PS mutations is more complicated in the course of AD pathogenesis, not the case of simple absence of γ-secretase activity. As PS mutations show an autosomal dominant inheritance trait in FAD pedigree, it is plausible that mutant PS plays a dominant-negative role in WT PS/γ-secretase complexes as shown previously (Heilig et al., 2013; Zhou et al., 2017). Future studies are needed to demonstrate that a physical proximity between WT and mutant PS/γ-secretase is detected in the same organelle from this point of view. Moreover, acute PS elimination did not increase tau phosphorylation in human cortical neurons (Fig. 5*E*), another AD pathologic hallmark, while such anomalies were observed in iPSC-derived neurons from AD patients (Israel et al., 2012; Ochalek et al., 2017). This suggests that tau phosphorylation requires accumulation of toxic Aβ species (Jin et al., 2011), leading to ultimate lesions such as synapse deficits and neuronal degeneration (Nieweg et al., 2015; Kouroupi et al., 2017; Gajera et al., 2019).

In summary, this study uncovered the distinct regulation of the PS/γ-secretase complex in iPSC-derived human neural cells compared with that revealed in previously reported mouse studies, which may underlie the failures of clinical trials for γ-secretase inhibitors. Despite the profound phenotypic differences of the PS/γ-secretase used in this study, however, the detailed molecular mechanisms underlying the substrate cleavage and subcellular localization of distinct PS/γ-secretases remain to be resolved. Furthermore, it would be much better to recapitulate our results in more suitable systems such as human brain organoids/spheroids, in which glial cell types can be developed concomitantly and interacted with neurons functionally (Paşca et al., 2015; Madhavan et al., 2018; Ormel et al., 2018). Using the novel cellular model in this study, future studies will clarify the causative molecular changes in PS/γ-secretase from physiological to pathophysiological states in the course of AD pathogenesis and lead to the development of novel therapeutic medicines.

## Synthesis

Reviewing Editor: Karl Herrup, University of Pittsburgh School of Medicine Decisions are customarily a result of the Reviewing Editor and the peer reviewers coming together and discussing their recommendations until a consensus is reached. When revisions are invited, a fact-based synthesis statement explaining their decision and outlining what is needed to prepare a revision will be listed below. The following reviewer(s) agreed to reveal their identity: Sarah Hernandez. Note: If this manuscript was transferred from JNeurosci and a decision was made to accept the manuscript without peer review, a brief statement to this effect will instead be what is listed below.

The manuscript by Watanabe et al. addresses an important problem that has gone un-solved in the Alzheimer’s disease research field: what are the exact substrate specificities of the gamma-secretase complexes formed by the two isoforms of presenilin (PSEN1 and PSEN2)? The authors use creative genetic strategies to examine substrate specificity and processing by PSEN1 and PSEN2 in human iPSC-derived cortical neurons. Through the use of conditional knockout iPSC lines, they examine the impact of single and double PSEN deletions on substrate (e.g., APP) processing and various parameters of neuronal differentiation. They then compare their results with previous findings in mouse models thus highlighting a major strength of this manuscript, namely, that the work was carried out in human cells.

The strengths of the work far outweigh its weakness. Nonetheless, in the spirit of producing the strongest possible work, the reviewers have cited a number of places where revision or clarification would be advantageous. You will note that virtually no new experiments are required (though one or two are suggested). The point-by-point reply to these concerns should indicate your response to each item and (where appropriate) the place in the manuscript where changes were made in response.

• The authors interpret their findings in Figure 3B-D to mean that iPSC-derived NPCs do not possess self-renewal capabilities in the absence of both PS1 and PS2. However, the results appear to only show a reduced capacity for NPC maintenance. The discussion of the findings should be tempered to be more accurate here. Alternatively, if they wish to conclude that self-renewal capacity is lost, the authors should assess NPC markers, as was done in panel A, after the second NS dissociation step. For example, RT-PCR or staining data of NPC markers after the second NS stage would offer more solid support the claims made.

• The authors interpret their findings in Figure 1C to mean that "APP possibly serves as a neuronal adhesive protein". This is plausible, but a bit overinterpreted given the absence of specific adhesion data. As only one alternative explanation, PSEN transcripts may have a longer half-life in this cell type.

• In Figure 4, the authors interpret their data to suggest that PS elimination does not influence neuronal differentiation. However, Cre was transduced 5 days after terminal differentiation, meaning expression of PS was only eliminated after cells had already begun the initial steps of differentiating into neurons. Viewed in this light, a more conservative analysis would be advised. For example, the data support the interpretation that PS2 specifically does not influence the later steps neuronal differentiation, and elimination of PS1 following terminal differentiation, in combination with PS2 loss, does not affect maintenance of the neuronal phenotype.

• One of the more intriguing findings of the work is that there are species-specific differences in the relationship of PSEN1/PS1 with Notch signaling. The authors’ interpretation is logically sound. They might wish to consider the alternative of cell-type specific contributions to organismal phenotypes. One reviewer noted that PS1 KO homozygous mice would have ubiquitous loss of PS1 in all cell types. While more complicated than the authors’ hypothesis, it is conceivable that the mouse in vivo data reflect indirect effects caused by PS1 deletion in other cell types. A mention of alternatives should be included in the Discussion

• The authors interpret their findings in Figure 5D to mean that N-cadherin is cleaved solely by PS1. However, the effect of N-cad cleavage appears to be potentiated by PS2 loss. Also, in Figure 5, it would help if there were a single graph (as in Figure 6C) that quantifies the Aβ42/Aβ40 ratio for each genetic permutation analyzed to show the extent to which these iPSC-derived cell lines possess AD-related pathological processing.

• One of the reviewers wondered whether the authors had attempted to document changes in cell structure following acute PSEN deletion. Did they note increases or decreases in the patterns of cellular projections as evidenced by morphology, dendritic branching or synaptic changes? A first level of inquiry could be simply western blots on synaptosome preparations or single synapse analysis like mass synaptometry.

• This same reviewer questioned whether there had been any effort made to note changes in total or pathological tau especially in their novel context of acute deletion of PSEN. This could be a strong point as it would stand in contrast to other literature reports that mostly look in constitutive KO cell models.

Both reviewers felt that the Discussion (and to a certain extent the Introduction) could be more thoroughly grounded in the existing literature.

• In the abstract the authors indicate that their findings explain how PS1/PS2 functions underlie AD pathogenesis. This is so, but unfortunately this important point is not explored in any depth in the discussion.

• Though this may be outside the scope of current study, one reviewer expressed the opinion that the discussion of the relationship of the current manipulations to the state of tau in the iPSC neurons would enrich the manuscript for specialized and general readers of journal and offered references A to D below as help with discussion.

• While the authors compare their work with previous mouse PS1 cKO studies, it would help the reader if the Discussion included how the previous cKOs were achieved and whether the analysis specifically included examination of cortical neurons. At a higher level of analysis, this same reviewer noted that while the authors show that PS1 KO in iPSC-derived cortical neurons does not compromise APP processing, it is nonetheless true that mutations in PS1 are a major cause (∼80%) of familial AD. This is an apparent disconnect that needs explanation.

• While the data surrounding the processing of Notch are solid, the contribution of other pathways such as SHH, BMP or Wnt cannot be ruled out. Cross talk or independent effects thereof could be entertained as possibilities. Thus, while the experimental design is fine, the Discussion could be expanded to include this possibility. Additional literature references for enhancing the context and perspective of this manuscript were suggested. (Ref E to H)

• This same reviewer, while recognizing the importance of doing these experiments in human cells, still felt that there were limitations that should be mentioned. As powerful as the iPSC system might be, cultured neuronal cells are still nowhere near the complex mature/aging neuron phenotype of cells in in human brain. The full diversity of cell types (e.g., glia or oligodendrocytes) is not represented, nor do the iPSC neurons have functional networks that mimic cognition in human. It is suggested to discuss the limitations inherent in this approach.

Clarification of Legends and Methods

• Please provide dilution factors used for immunocytochemistry and Western blots in the methods section.

• Please update the methods section to include the protocol for flow cytometry and include flow cytometry plots in supplemental data.

• The Figure legends indicate number of "different cultures" used per experiment, however it is not clear if these are technical or biological replicates. Please revise.

• The legend for Figure 4A should include when during the differentiation process the were taken.

• Define "nc" in the legends of figures that use this nomenclature, i.e. Figure 2.

• In Figure 4, please identify which band on the Western blot is APP

• In Figure 4B, the levels of PS1 appear to be decreasing over time where levels of PS2 appear to be increasing over time in ΔCre samples. If this is an expected result please add thoughts on these findings in the text.

• Typo on page 34 line 6 - inclusion of an additional "with".

References:

A) Neurons derived from sporadic Alzheimer’s disease iPSCs reveal elevated TAU hyperphosphorylation, increased amyloid levels, and GSK3B activation. Alzheimers Res Ther. 2017; 9: 90. Published online 2017 Dec 1. doi: 10.1186/s13195-017-0317-z

B) Alzheimer’s disease-related amyloid-β induces synaptotoxicity in human iPS cell-derived neurons. Cell Death Dis. 2015 Apr; 6(4): e1709.

Published online 2015 Apr 2. doi: 10.1038/cddis.2015.72

C) Mass synaptometry: High-dimensional multi parametric assay for single synapses. J Neurosci Methods. 2019 Jan 15;312:73-83. doi: 10.1016/j.jneumeth.2018.11.008. Epub 2018 Nov 20

D) Defective synaptic connectivity and axonal neuropathology in a human iPSC-based model of familial Parkinson’s disease, PNAS May 2, 2017 114 (18) E3679-E3688; first published April 17, 2017; https://doi.org/10.1073/pnas.1617259114

E) Integration of BMP, Wnt, and Notch signaling pathways in osteoblast differentiation. ) J Cell Biochem. J Cell Biochem. 2011 Dec; 112(12): 3491-3501.

F) LRP2 in ependymal cells regulates BMP signaling in the adult neurogenic niche. J Cell Sci. 2010 Jun 1;123(Pt 11):1922-30. doi: 10.1242/jcs.065912. Epub 2010 May 11.

G) Escape from Pluripotency via Inhibition of TGF-β/BMP and Activation of Wnt Signaling Accelerates Differentiation and Aging in hPSC Progeny Cells. Stem Cell Reports

. 2017 Nov 14;9(5):1675-1691. doi: 10.1016/j.stemcr.2017.09.024.

H) Modeling sporadic ALS in iPSC-derived motor neurons identifies a potential therapeutic agent. Nat Med. 2018 Oct;24(10):1579-1589. doi: 10.1038/s41591-018-0140-5.
