# Supplementary figures and images for: Flexible and Accurate Substrate Processing with Distinct Presenilin/γ-Secretases in Human Cortical Neurons
Source: eNeuro. 2021 Mar 2;8(2):ENEURO.0500-20.2021. doi: 10.1523/ENEURO.0500-20.2021 (PMC7932187; doi:10.1523/ENEURO.0500-20.2021)

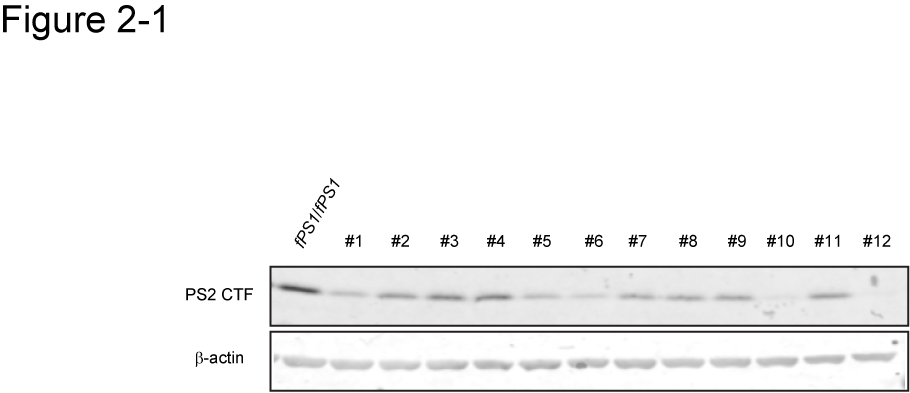

Supplement: Extended Data Figure 2-1 — PS2 Western blot analysis of single clones transfected with PS2 sgRNA and Cas9 proteins. Two clones #10 and #12 were nearly negative for PS2 proteins. Download Figure 2-1, TIF file. [file enu-eN-NWR-0500-20-s01.tif]

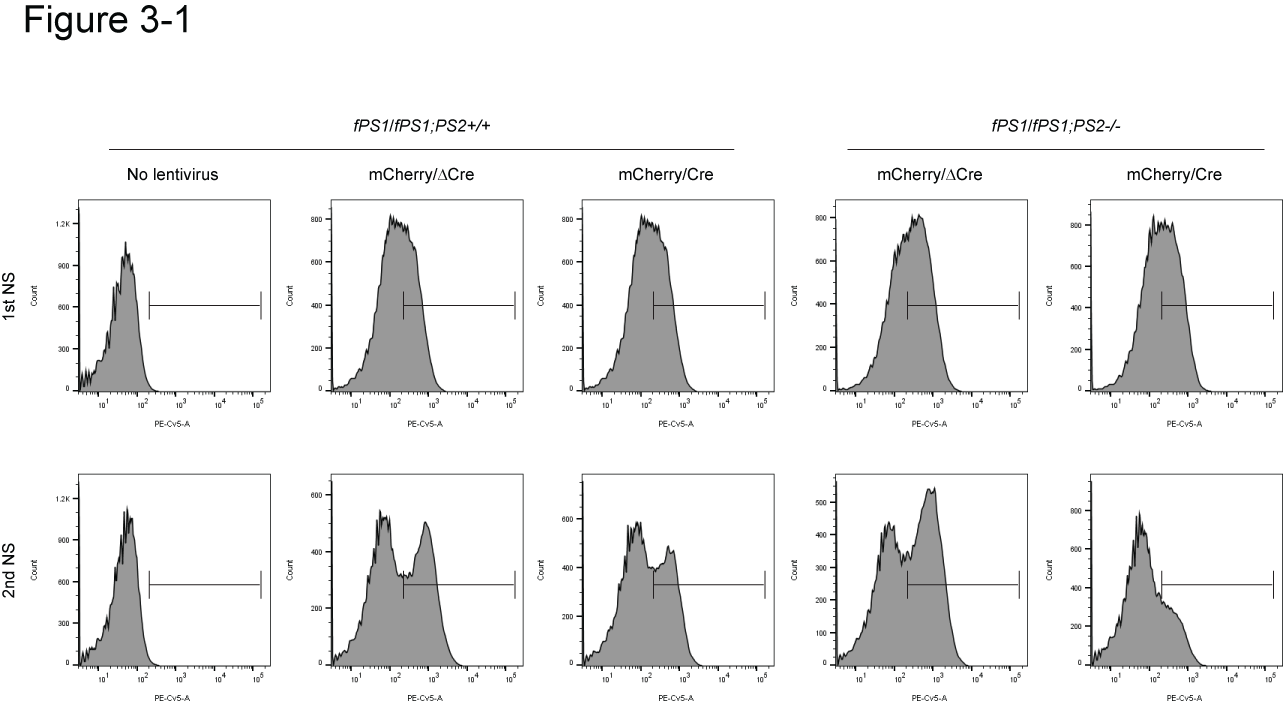

Supplement: Extended Data Figure 3-1 — Flow cytometry analysis in colony forming assay. Dissociated neurospheres were fractionated by single cell gate and analyzed with mCherry fluorescence. The representative histograms were shown from both primary and secondary NS analyses. Note that mCherry fluorescence got stronger in secondary NS due to a delayed expression from lentivirus in primary NS. Download Figure 3-1, TIF file. [file enu-eN-NWR-0500-20-s02.tif]

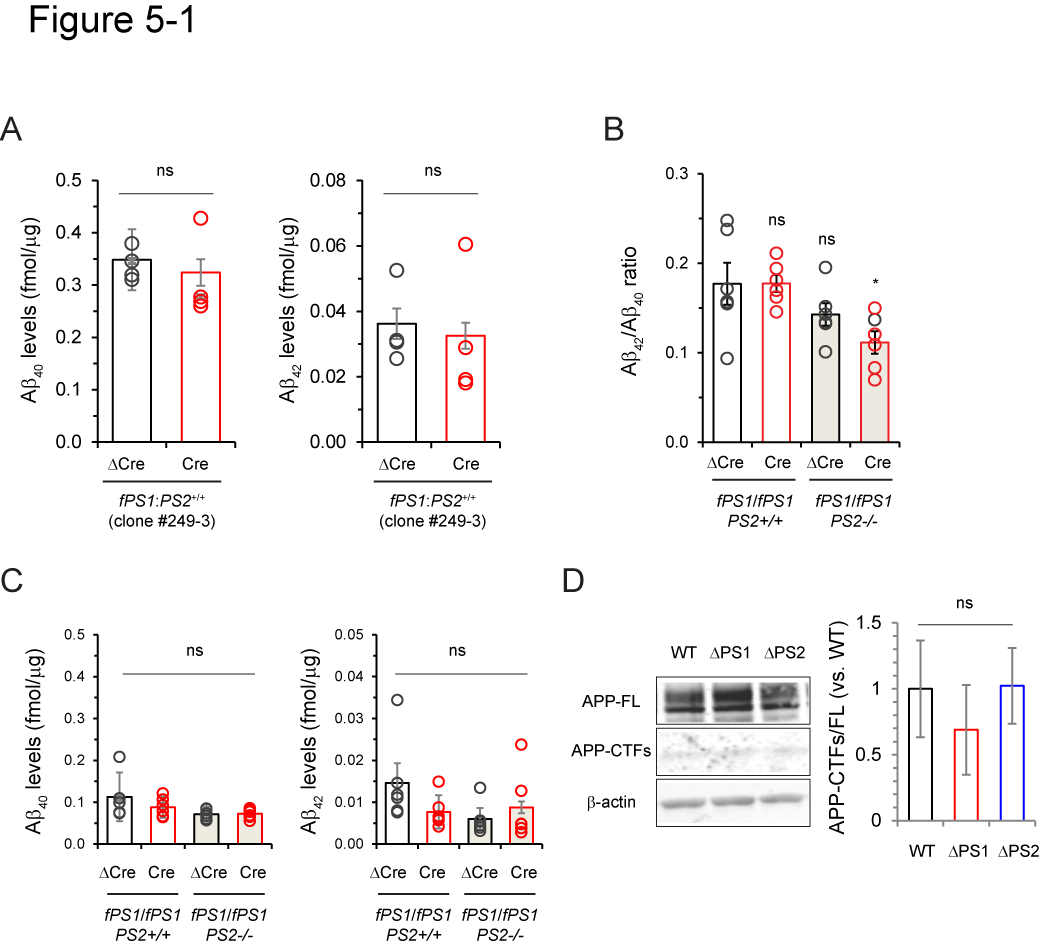

Supplement: Extended Data Figure 5-1 — Processing of APP in the PS-null iPSC-derived neurons. A, ELISA measurement specific for Aβ40 and Aβ42 in the iPSC-neurons from another fPS1/fPS1;PS2+/+ clone (#249-3) infected with ΔCre or Cre lentivirus. Data represent the mean ± SEM (n = 3 of independent culture batches). ns, not significant by Student’s t test between the genotypes. B, Calculated Aβ42/Aβ40 ratio was also drawn from the data in Figure 5A. Data represent the mean ± SEM (n = 3 of independent culture batches). ns, not significant; *p < 0.05 by Dunnett’s test versus the control. C, ELISA measurement specific for Aβ40 and Aβ42 in the iPSC-neurons of fPS1/fPS1;PS2+/+ and fPS1/fPS1;PS2-/- infected with ΔCre or Cre lentivirus, with DAPT treatment for 48 h. Data represent the mean ± SEM (n = 4–5 of independent culture batches). ns, not significant by Dunnett’s test versus the control. D, Levels of the APP-CTFs are quantified in WT, ΔPS1, and ΔPS2 neurons by Western blotting. No alteration of APP-CTFs was observed among the genotypes. Data represent the mean ± SEM (n = 3–4 of independent culture batches). ns, not significant by Dunnett’s test versus the control. Download Figure 5-1, TIF file. [file enu-eN-NWR-0500-20-s03.tif]

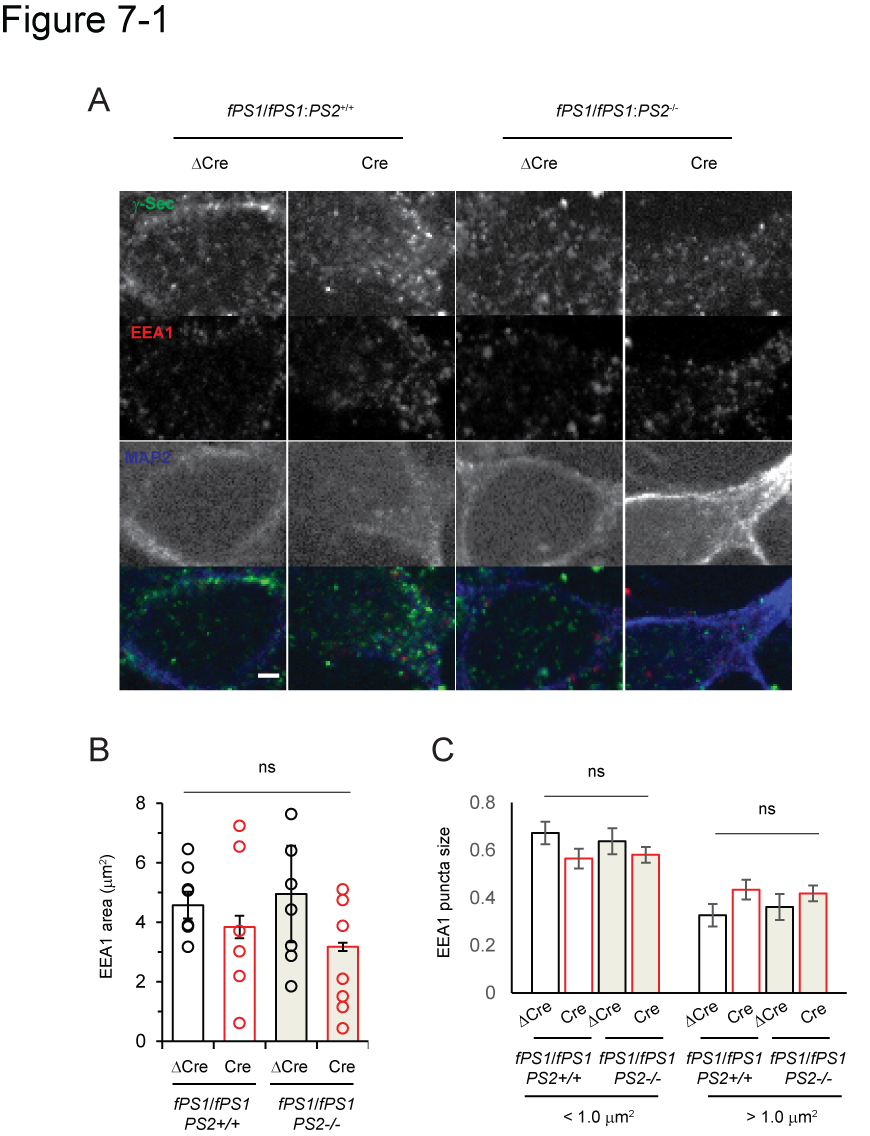

Supplement: Extended Data Figure 7-1 — No significant change of EEA1+ puncta in the iPSC-neurons of fPS1/fPS1;PS2+/+ and fPS1/fPS1;PS2-/- infected with ΔCre or Cre lentivirus. A, Representative images of iPSC-neurons stained with antibodies specific for γ-secretase complexes (A5226A), EEA1, and MAP2 are shown. There are many puncta of γ-secretase complexes and EEA1 in perinuclear region and neurites. Scale bar: 2 μm. B, Quantification of EEA1+ puncta area in the iPSC-neurons, and no significant difference between the genotypes. Data represent the mean ± SEM (n = 4–6 of independent culture batches). ns, not significant by Dunnett’s test versus the control. C, Size distribution of EEA1+ puncta of iPSC-derived neurons with the threshold of 1.0-μm2 puncta. Data represent the mean ± SEM (n = 4–6 of independent culture batches). ns, not significant by Dunnett’s test versus the control. Download Figure 7-1, TIF file. [file enu-eN-NWR-0500-20-s04.tif]
